# On Quantum Ergodicity for Higher Dimensional Cat Maps

**DOI:** 10.1007/s00220-025-05350-1

**Published:** 2025-07-02

**Authors:** Pär Kurlberg, Alina Ostafe, Zeev Rudnick, Igor E. Shparlinski

**Affiliations:** 1 Department of Mathematics, Royal Institute of Technology, 100 44 Stockholm, Sweden; 2 School of Mathematics and Statistics, University of New South Wales, Sydney, NSW 2052 Australia; 3 School of Mathematical Sciences, Tel-Aviv University, 69978 Tel-Aviv, Israel

## Abstract

We study eigenfunction localization for higher dimensional cat maps, a popular model of quantum chaos. These maps are given by linear symplectic maps in $\operatorname {Sp}(2g,{{\mathbb {Z}}})$, which we take to be ergodic. Under some natural assumptions, we show that there is a density one sequence of integers *N* so that as *N* tends to infinity along this sequence, all eigenfunctions of the quantized map at the inverse Planck constant *N* are uniformly distributed. For the two-dimensional case ($g=1$), this was proved by Kurlberg and Rudnick (Duke Math J 103:47–78, 2000). The higher dimensional case offers several new features and requires a completely different set of tools, including from additive combinatorics, such as a bound of Bourgain (J Am Math Soc 18:477–499, 2005) for Mordell sums, and a study of tensor product structures for the cat map, which has never been exploited in this context.

## Introduction

### Quantum ergodicity and the quantized cat map

Eigenfunction localization is one of the central topics of Quantum Chaos. In this paper, we examine this question for an important “toy model”, the quantized cat map [12], aiming for higher dimensional maps. Our techniques, after a preliminary reduction, combine analytic number theory and additive combinatorics.

Denote by $\operatorname {Sp}(2g,{{\mathbb {Z}}})$ the group of all integer matrices *A* which preserve the symplectic form

1.1 $$\begin{aligned} \omega (\textbf{x},\textbf{y}) = \textbf{x}_1 \cdot \textbf{y}_2-\textbf{x}_2\cdot \textbf{y}_1, \end{aligned}$$

with $\textbf{x}=(\textbf{x}_1,\textbf{x}_2)$, $\textbf{y}=(\textbf{y}_1,\textbf{y}_2)\in {{\mathbb {R}}}^g\times {{\mathbb {R}}}^g$. Any $A\in \operatorname {Sp}(2g,{{\mathbb {Z}}})$ generates a classical dynamical system via its action on the torus ${\mathbb {T}}^{2g}= {{\mathbb {R}}}^{2g}/{{\mathbb {Z}}}^{2g}$. We say that this dynamical system is ergodic if for almost all initial positions $\textbf{x}\in {\mathbb {T}}^{2g}$, the orbit $\{A^j\textbf{x}:~j\ge 0\}$ is uniformly distributed in ${\mathbb {T}}^{2g}$. This is equivalent to *A* having no eigenvalues which are roots of unity, see [11].

Associated to any $A\in \operatorname {Sp}(2g,{{\mathbb {Z}}})$ is a quantum mechanical system. We briefly recall the key definitions: One constructs for each integer $N\ge 1$ (the inverse Planck constant, necessarily an integer here) a Hilbert space of states ${{{\mathcal {H}}}}_{N}= L^2(({{\mathbb {Z}}}/N{{\mathbb {Z}}})^g)$ equipped with the scalar product

$$\begin{aligned} \langle \varphi _1, \varphi _2 \rangle = \frac{1}{N^g} \sum _{\textbf{u} \in ({{\mathbb {Z}}}/N{{\mathbb {Z}}})^g} \varphi _1(\textbf{u}) \overline{ \varphi _2(\textbf{u})}, \qquad \varphi _1, \varphi _2 \in {{{\mathcal {H}}}}_{N}. \end{aligned}$$

The basic observables are given by the unitary operators

$$\begin{aligned} \operatorname {T}_N(\vec {n}):{{{\mathcal {H}}}}_{N}\rightarrow {{{\mathcal {H}}}}_{N}, \qquad \vec {n}=(\vec {n}_1, \vec {n}_2)\in {{\mathbb {Z}}}^g\times {{\mathbb {Z}}}^g={{\mathbb {Z}}}^{2g}, \end{aligned}$$

as follows

1.2 $$\begin{aligned} \left( \operatorname {T}_N(\vec {n})\varphi \right) (\textbf{Q}) = {\,{\textbf{e}}}_{2N}(\vec {n}_1\cdot \vec {n}_2){\,{\textbf{e}}}_N(\vec {n}_2\cdot \textbf{Q}) \varphi (\textbf{Q}+\vec {n}_1), \end{aligned}$$

where hereafter we always follow the convention that integer arguments of functions on ${{\mathbb {Z}}}/N{{\mathbb {Z}}}$ are reduced modulo *N* (that is, $\varphi (\textbf{Q}+\vec {n}_1) = \varphi (\textbf{Q}+(\vec {n}_1 \bmod N))$). It is also easy to verify that (1.2) implies

$$\begin{aligned} \operatorname {T}_N({\textbf{m}}) \operatorname {T}_N(\vec {n}) = {\,{\textbf{e}}}_{2N}\left( \omega \left( {\textbf{m}},\vec {n}\right) \right) \operatorname {T}_N({\textbf{m}}+\vec {n}), \end{aligned}$$

where $\omega \left( {\textbf{m}},\vec {n}\right)$ is defined by (1.1) and

$$\begin{aligned} {\,{\textbf{e}}}(z) = \exp \left( 2\pi i z\right) ,\quad {\mathbf {\,e}}_k(z)={\,{\textbf{e}}}(z/k), \end{aligned}$$

see also [19, Equation (2.6)].

For each real-valued function $f\in C^\infty ({\mathbb {T}}^{2g})$ (an “observable”), one associates a self-adjoint operator $\operatorname {Op}_N(f)$ on ${{{\mathcal {H}}}}_{N}$, analogous to a pseudo-differential operator with symbol *f*, defined by

1.3 $$\begin{aligned} \operatorname {Op}_{N}(f) = \sum _{\vec {n}\in {{\mathbb {Z}}}^{2g}}{{\widehat{f}}}(\vec {n}) \operatorname {T}_N(\vec {n}), \end{aligned}$$

where

1.4 $$\begin{aligned} f(\textbf{x})= \sum _{\vec {n}\in {{\mathbb {Z}}}^{2g}} {{\hat{f}}}(\textbf{n}){\,{\textbf{e}}}(\textbf{n} \cdot \textbf{x}). \end{aligned}$$

Assuming $A=I\bmod 2$ (this condition can be weakened, see, for example, the definition of the subgroup $\operatorname {Sp}_\vartheta (2g, {{\mathbb {Z}}})$ of $\operatorname {Sp}(2g, {{\mathbb {Z}}})$ as in [16, p. 817], where *d* is used instead of *g*),

for each value of the inverse Planck constant $N\ge 1$, there is a unitary operator $U_N(A)$ on ${{{\mathcal {H}}}}_{N}$, unique up to scalar multiples, which generates the quantum evolution, in the sense that for every observable $f\in C^\infty ({\mathbb {T}}^{2g})$, we have the exact Egorov property

1.5 $$\begin{aligned} U_N(A)^*\operatorname {Op}_N(f) U_N(A) = \operatorname {Op}_N(f\circ A) , \end{aligned}$$

where $U_N(A)^*= \overline{U_N(A)}^{\,t}$, we refer to [18, 23] for a detailed exposition in the case $g=1$ and [16] for higher dimensions.

The stationary states of the system are the eigenfunctions of $U_N(A)$ and one of the main goals is to study their localization properties. In particular, given any normalized sequence of eigenfunctions $\psi _N\in {{{\mathcal {H}}}}_{N}$, we ask if the expected values of observables in these eigenfunctions converge, as $N\rightarrow \infty$, to the classical average (see § 2.1 for precise definitions), that is, that

1.6 $$\begin{aligned} \lim _{N\rightarrow \infty } \langle \operatorname {Op}_N(f)\psi _N,\psi _N \rangle = \int _{{\mathbb {T}}^{2g}} f(\textbf{x})d\textbf{x} \end{aligned}$$

for all $f\in C^\infty ({\mathbb {T}}^{2g})$, in which case we say that the sequence of eigenfunctions $\{\psi _N\}$ is uniformly distributed.

A fundamental result is the Quantum Ergodicity Theorem [4, 25, 28], valid in great generality, which in our setting asserts that if *A* is ergodic, then for any **orthonormal basis** $\varPsi _N = \{\psi _{j,N}:~j=1,\ldots ,N^g\}$ of eigenfunctions of $U_N(A)$ in ${{{\mathcal {H}}}}_{N}$, there is a subset ${{\mathcal {S}}}\subseteq \{1,\ldots ,N^g\}$ with asymptotic density one (that is, $\sharp {{\mathcal {S}}}/N^g\rightarrow 1$, where $\sharp {{\mathcal {S}}}$ denotes the cardinality of ${{\mathcal {S}}}$) so that $\psi _{j,N}$ are uniformly distributed for all $j\in {{\mathcal {S}}}$, see [3]. If *all* eigenfunctions are uniformly distributed, the system is said to exhibit Quantum Unique Ergodicity [24]. In fact, more generally,

setting

$$\begin{aligned} \Delta _A(f,N) = \max _{\psi _N,\psi '_N } \left| \langle \operatorname {Op}_N(f)\psi _{ N}, \psi _{N}' \rangle -\langle \psi _N,\psi _N'\rangle \int _{{\mathbb {T}}^{2g}} f({\textbf{x}})d{\textbf{x}}\right| , \end{aligned}$$

the maximum taken over all pairs of normalized eigenfunctions $\psi _N,\psi '_N$ of $U_N(A)$, we ask if for all $f\in C^\infty ({\mathbb {T}}^{2g})$,

1.7 $$\begin{aligned} \lim _{\begin{array}{c} N\rightarrow \infty \\ N \in {{\mathcal {N}}} \end{array}} \Delta _A(f,N) = 0 , \end{aligned}$$

where ${{\mathcal {N}}}$ is a set of integers of asymptotic density 1 (that is, $\# ({{\mathcal {N}}}\cap [1,x]) = x + o(x)$ as $x \rightarrow \infty$).

#### Remark 1.1

It is interesting to note that even if we are mainly interested in scarring (that is, decay of diagonal matrix coefficients corresponding to $\psi _{N}' = \psi _{N}$ in the above definition of $\Delta _A(f,N)$ and establishing (1.6)), for the full argument we still need estimates for off-diagonals coefficients of the “nontrivial” tensor component in § 6.4.

The two-dimensional ($g=1$) cat map is where the first counterexamples (“scars”) to QUE have been proved to exist [9], associated with the *N*, where the period $\operatorname {ord}(A,N)$ of the classical map reduced modulo *N* was almost minimally small, about $2 \log N/\log \lambda$, where $\lambda >1$ is the largest eigenvalue of *A*. We note that the relevance of the classical period to the quantum system was recognized early on in the theory [5, 12, 15]. In [19], it was shown that if $\operatorname {ord}(A,N)$ was somewhat larger than $N^{1/2}$ (and *N* satisfies a further genericity condition), then *all* eigenfunctions in ${{{\mathcal {H}}}}_{N}$ are uniformly distributed. Note that the condition holds for almost all primes [7]. Separately, it was shown that $\operatorname {ord}(A,N)$ is sufficiently large for almost all integers *N*.

A breakthrough was made by Bourgain [2], who showed that when $N=p$ is prime (that, and the prime power, cases are the basic building block for the theory since the quantization with respect to composite moduli arise as tensor products of quantizations with respect to prime power moduli), for all eigenfunctions to be uniformly distributed it suffices to take $\operatorname {ord}(A,p)>p^\varepsilon$, for some $\varepsilon >0$, a condition that is much easier to establish than a bound bigger than $p^{1/2}$. This allowed Bourgain [2] to give a polynomial rate of convergence for a version of (1.7) over a sequence of almost all integers: for some $\delta >0$, for almost all *N* we have $\Delta _A(f,N) \leqslant N^{-\delta }$. Using a different approach, in [20] it is shown that one can take any $\delta < 1/60$.

### Higher dimensional cat maps

Higher dimensional cat maps offer several more challenges. In particular, we address the analogue of [19], namely all eigenfunctions in ${{{\mathcal {H}}}}_{N}$ being uniformly distributed for almost all integers *N*. We do not discuss other aspects of localizations, such as entropy bounds [8, 22] and showing that all semiclassical measures have full support [6, 17, 26].

In higher dimensions (that is, for $g>1$), there is a significant change. Kelmer [16] has shown that if *A* has nontrivial invariant rational istropic subspaces, then for all *N*, uniform distribution (1.7) fails – there are so-called scars.

So we assume that there are no nontrivial invariant rational isotropic subspaces. We want to find a full density sequence ${\mathcal {N}}$ of integers *N* for which *all* eigenfunctions of $U_N(A)$ are uniformly distributed, that is, if we fix $f\in C^\infty ({\mathbb {T}}^{2g})$ then we have (1.7). If this holds for all *f* then we say that *A* *satisfies QUE for the subsequence* ${\mathcal {N}}$.

Recall that we assume ergodicity, equivalently, that the eigenvalues of $A\in \operatorname {Sp}(2g,{{\mathbb {Z}}})$ are not roots of unity. For our results, we need to impose a further condition on *A*, that no ratio of distinct eigenvalues is a root of unity. In addition, we assume that the characteristic polynomial $f_A(x)=\det (xI-A)\in {{\mathbb {Z}}}[x]$ is separable (that is, has no multiple roots)

Our main result establishes (1.7) for almost all integers under the above conditions on *A*:

#### [Style2 Style2]Theorem 1.2

Let $A\in \operatorname {Sp}(2g,{{\mathbb {Z}}})$, with a separable characteristic polynomial, be such that no ratio of distinct eigenvalues is a root of unity. Assume further that there are no nontrivial *A*-invariant rational isotropic subspaces. Then *A* satisfies QUE as in (1.7) for some set ${{\mathcal {N}}}$ of asymptotic density 1.

One can show that if the characteristic polynomial of *A* is irreducible, then there are no nontrivial *A*-invariant rational subspaces.

### Plan of the proof

We establish Theorem 1.2 via the following sequences of steps.

(i) To prove (1.7), it suffices to show it for the basic observables (translation operators) $\operatorname {T}_N(\vec {n}) = \operatorname {Op}_N(f)$, $f({\textbf{x}})= {\,{\textbf{e}}}\left( {\textbf{x}}\cdot \vec {n}\right)$ (see also (1.3)), with frequency $\vec {n}$ growing slowly with *N*.
  Assume that the characteristic polynomial $f_A(x)=\det (xI-A)$ is irreducible over the rationals. Then we reduce the problem of estimating high powers $\Big | \langle \operatorname {T}_N(\vec {n})\psi ,\psi ' \rangle \Big |^{4\nu }$ of the matrix elements for all normalized eigenfunctions $\psi , \psi '$, to a problem of estimating the number of solutions to the matrix congruence
  $$\begin{aligned} A^{k_1}+\ldots +A^{k_{2\nu }} -A^{\ell _1} -\ldots -A^{\ell _{2\nu }} \equiv O\bmod N, \end{aligned}$$
  for the zero matrix *O* with $1\leqslant k_i,\ell _i \leqslant \operatorname {ord}(A,N)$, $i =1, \ldots , 2\nu$, see Lemmas 4.1 and 4.3 (since indeed $f_A(x)$ being irreducible implies there is no nontrivial zero-divisor in ${{\mathbb {Q}}}^{2g}$).
(ii) In turn, this number can be treated by exponential sums. However this reduction does not work directly due to the lack of nontrivial bounds on such sums except when $N=p$ is a prime, modulo which the characteristic polynomial of *A* splits completely, in which case we can apply a striking result of Bourgain [1] on short “Mordell sums". The result, roughly speaking, is that there is some $\gamma >0$ so that for almost all split primes *p*,
  1.8 $$\begin{aligned} \max _{\psi , \psi ' } \left| \langle \operatorname {T}_p (\vec {n})\psi ,\psi ' \rangle \right| \leqslant p^{-\gamma }. \end{aligned}$$
(iii) To take advantage of the bound (1.8) for split primes, we prove that the operators $\operatorname {T}_N(\vec {n})$ have a tensor product structure with respect to the Chinese Remainder Theorem, however with some losses depending on certain greatest common divisors. Thus we deal with the operators $\operatorname {T}_p(\vec {n})$ via exponential sums and use the trivial bound
  $$\begin{aligned} \left| \langle \operatorname {T}_M(\vec {n})\psi ,\psi ' \rangle \right| \leqslant 1, \end{aligned}$$
  where *M* is the largest divisor of *N* without split prime factors.
(iv) Finally, using some results from the *anatomy of integers* (§ 7) we show that for almost all integers *N*, the saving we obtain from the split primes $p\mid N$, exceeds the losses we incur in our version of the Chinese Remainder Theorem.
(v) When the characteristic polynomial $f_A(x)$ of *A* is reducible, but separable, we require extra consideration, as the reduction to counting solutions of matrix congruences fails when $\vec {n}$ is a non-trivial zero-divisor. We make use of an additional tensor structure to reduce to the setting of congruences for a smaller dimensional case, see § 6 for details.

### Notation

Throughout the paper, the notations

$$\begin{aligned}X = O(Y), \qquad X \ll Y, \qquad Y \gg X \end{aligned}$$

are all equivalent to the statement that the inequality $|X| \leqslant c Y$ holds with some constant $c> 0$, which may depend on the matrix *A*, and occasionally, where obvious also on the real parameter $\varepsilon$.

We recall that the additive character with period 1 is denoted by

$$\begin{aligned} z \in {\mathbb {R}}\ \mapsto \ {\,{\textbf{e}}}(z) = \exp \left( 2\pi i z\right) . \end{aligned}$$

For an integer $k \geqslant 1$ it is also convenient to define

$$\begin{aligned} {\mathbf {\,e}}_k(z) = {\,{\textbf{e}}}(z/k). \end{aligned}$$

The letter *p*, with or without indices, always denotes prime numbers.

Given an algebraic number $\gamma$ we denote by $\operatorname {ord}(\gamma , N)$ its order modulo *N* (assuming that the ideals generated by $\gamma$ and *N* are relatively prime in an appropriate number field). In particular, for an element $\lambda \in {{\mathbb {F}}}_{p^s}$, $\operatorname {ord}(\lambda ,p)$ represents the order of $\lambda$ in ${{\mathbb {F}}}_{p^s}$.

Similarly, we use $\operatorname {ord}(A,N)$ to denote the order of *A* modulo *N* (which always exists if $\gcd (\det A, N)= 1$ and in particular for $A\in \operatorname {Sp}(2g,{{\mathbb {Z}}})$).

For a finite set ${{\mathcal {S}}}$ we use $\sharp {{\mathcal {S}}}$ to denote its cardinality.

As usual, we say that a certain property holds for *almost all* elements of an infinite sequence $s_n$, $n =1,2, \ldots$, if it fails for *o*(*x*) terms with $n \leqslant x$, as $x \rightarrow \infty$. In particular, we say that it holds for *almost all* primes *p* and positive integers *N* if for $x \rightarrow \infty$, it fails for $o(x/\log x)$ primes $p\leqslant x$ and *o*(*x*) positive integers integers $N\leqslant x$, respectively.

Similarly, we say that a certain property holds for *a positive proportion* of primes *p* or, equivalently for a set of *positive density*, if for some constant $c>0$, which throughout this work may depend on the matrix *A*, for all sufficiently large *x* it holds for at least $c x/\log x$ primes $p\leqslant x$.

## A Chinese Remainder Theorem for the Operators $\operatorname {T}_N(\vec {n})$

### Observables

We begin by defining the mixed translation operators. Given $r\in {{\mathbb {Z}}}$, coprime to *N*, and $\vec {n}=(\vec {n}_1, \vec {n}_2)\in {{\mathbb {Z}}}^g\times {{\mathbb {Z}}}^g={{\mathbb {Z}}}^{2g}$, we define a unitary operator $\operatorname {T}_N^{(r)}(\vec {n}):{{{\mathcal {H}}}}_{N}\rightarrow {{{\mathcal {H}}}}_{N}$ by

$$\begin{aligned} \left( \operatorname {T}_N^{(r)}(\vec {n})\varphi \right) (\textbf{Q}) = {\,{\textbf{e}}}_{2N}(r \vec {n}_1\cdot \vec {n}_2){\,{\textbf{e}}}_N(r \vec {n}_2\cdot \textbf{Q}) \varphi (\textbf{Q}+\vec {n}_1). \end{aligned}$$

We have

2.1 $$\begin{aligned} \operatorname {T}_N^{(r)}({\textbf{m}}) \operatorname {T}_N^{(r)}(\vec {n}) = {\,{\textbf{e}}}_{2N}\left( r\omega \left( {\textbf{m}},\vec {n}\right) \right) \operatorname {T}_N^{(r)}({\textbf{m}}+\vec {n}), \end{aligned}$$

where $\omega \left( {\textbf{m}},\vec {n}\right)$ is defined by (1.1). In particular, taking powers gives

$$\begin{aligned} \left( \operatorname {T}_N^{(r)}\left( \vec {n}\right) \right) ^k = \operatorname {T}_N^{(r)}(k \vec {n}). \end{aligned}$$

The canonical commutation relations can be encapsulated in the relations

$$\begin{aligned} \operatorname {T}_N^{(r)}(\vec {n}) \operatorname {T}_N^{(r)}({\textbf{m}})={\,{\textbf{e}}}_N\left( r\omega \left( \vec {n},{\textbf{m}}\right) \right) \operatorname {T}_N^{(r)}({\textbf{m}}) \operatorname {T}_N^{(r)}(\vec {n}) \end{aligned}$$

and

$$\begin{aligned} ( \operatorname {T}_N^{(r)}(\vec {n}))^N = \operatorname {T}_N^{(r)}(N\vec {n}) = (-1)^{r N\vec {n}_1 \cdot \vec {n}_2} I, \quad \vec {n}=(\vec {n}_1,\vec {n}_2). \end{aligned}$$

For each function $f\in C^\infty ({\mathbb {T}}^{2g})$ on the classical phase space (an “observable”), one associates an operator $\operatorname {Op}_{N,r}(f)$ on ${{{\mathcal {H}}}}_{N}$, analogous to a pseudo-differential operator with symbol *f*, by

$$\begin{aligned} \operatorname {Op}_{N,r}(f) = \sum _{\vec {n}\in {{\mathbb {Z}}}^{2g}}{{\widehat{f}}}(\vec {n}) \operatorname {T}_N^{(r)}(\vec {n}), \end{aligned}$$

where ${{\widehat{f}}}(\vec {n})$ are defined by (1.4). If *f* is real valued, then $\operatorname {Op}_{N,r}(f)$ is self-adjoint.

When $r=1$, we recover the definitions of $\operatorname {T}_N= \operatorname {T}_N^{(1)}$ and $\operatorname {Op}_N= \operatorname {Op}_{N,1}$ in (1.2) and (1.3), respectively.

Let $A\in \operatorname {Sp}(2g,{{\mathbb {Z}}})$, satisfying the parity condition $A = I\bmod 2$. Fix $N\ge 1$ and *r* coprime to *N*. Then there is a unitary operator $U_{N,r}(A):~{{{\mathcal {H}}}}_{N}\rightarrow {{{\mathcal {H}}}}_{N}$, unique up to a scalar multiple, so that we have the exact Egorov property

2.2 $$\begin{aligned} U_{N,r}(A)^* \operatorname {T}_N^{(r)}(\vec {n}) U_{N,r}(A) =\operatorname {T}_N^{(r)}(\vec {n}A), \end{aligned}$$

fo all $\vec {n}\in {{\mathbb {Z}}}^{2g}$, which is a full analogue of (1.5).

### The Chinese Remainder Theorem and a tensor product structure

Assume that the inverse Planck constant *N* factors as $N=N_1 \cdot N_2$ with $N_1,N_2$ coprime. We then use the Chinese Remainder Theorem $\iota : {{\mathbb {Z}}}/N{{\mathbb {Z}}}\cong {{\mathbb {Z}}}/N_1{{\mathbb {Z}}}\oplus {{\mathbb {Z}}}/N_2{{\mathbb {Z}}}$ to get an isomorphism

$$\begin{aligned} \iota ^*: L^2(({{\mathbb {Z}}}/N_1{{\mathbb {Z}}})^g)\otimes L^2(({{\mathbb {Z}}}/N_2{{\mathbb {Z}}})^g) = \mathcal {{\mathcal {H}}}_{N_1} \otimes \mathcal {{\mathcal {H}}}_{N_2}\cong {{{\mathcal {H}}}}_{N}= L^2(({{\mathbb {Z}}}/N{{\mathbb {Z}}})^g) \end{aligned}$$

so that

2.3 $$\begin{aligned} \iota ^*(\varphi _1\otimes \varphi _2)(\textbf{Q}) = \varphi _1(\textbf{Q} \bmod N_1) \cdot \varphi _2(\textbf{Q} \bmod N_2) . \end{aligned}$$

The tensor product ${\mathcal {H}}_{N_1} \otimes {\mathcal {H}}_{N_2}$ carries the inner product

$$\begin{aligned} \Vert \varphi _1\otimes \varphi _2\Vert =\Vert \varphi _1\Vert \cdot \Vert \varphi _2\Vert \end{aligned}$$

and $\iota ^*$ is actually an isometry, because it maps the orthonormal basis of tensor products of normalized delta functions to normalized delta functions:

$$\begin{aligned} \iota ^* \left( N_1^{g/2}\delta _{\textbf{u}} \otimes N_2^{g/2} \delta _{\textbf{v}} \right) = N^{g/2} \delta _{\textbf{w}} \end{aligned}$$

where $$\textbf{w}= \textbf{u} \bmod N_1$$, $$\textbf{w} = \textbf{v} \bmod N_2$$.

Assume $N=N_1 \cdot N_2$ with $N_1>1$, $N_2>1$ coprime. Fix nonzero $r_1,r_2 \in {{\mathbb {Z}}}$ so that

2.4 $$\begin{aligned} N_2 r_2 + N_1 r_1=1. \end{aligned}$$

Necessarily $r_2$ is coprime to $N_1$ and $r_1$ is coprime to $N_2$.

#### [Style2 Style2 Style2]Lemma 2.1

For $\vec {n}=(\vec {n}_1, \vec {n}_2)\in {{\mathbb {Z}}}^g\times {{\mathbb {Z}}}^g$, the mixed translation operator $\operatorname {T}_N(\vec {n})=\operatorname {T}_N^{(1)}(\vec {n})$ is mapped, via the isomorphism $\iota ^*$, to $\operatorname {T}_{N_1}^{(r_2)}(\vec {n})\otimes \operatorname {T}_{N_2}^{(r_1)}(\vec {n})$:

$$\begin{aligned} \operatorname {T}_N(\vec {n}) \iota ^*\left( \varphi _1\otimes \varphi _2 \right) = \iota ^*\left( \left( \operatorname {T}_{N_1}^{(r_2)}(\vec {n})\varphi _1 \right) \otimes \left( \operatorname {T}_{N_2}^{(r_1)}(\vec {n})\varphi _2 \right) \right) . \end{aligned}$$

#### Proof

Inserting (2.4) gives

$$\begin{aligned} {\,{\textbf{e}}}_{2N}(\vec {n}_1\cdot \vec {n}_2)&= {\,{\textbf{e}}}\left( \frac{(N_2 r_2 + N_1 r_1)\vec {n}_1\cdot \vec {n}_2}{2N_1N_2}\right) \\&= {\,{\textbf{e}}}\left( \frac{r_2 \vec {n}_1\cdot \vec {n}_2}{2N_1}\right) {\,{\textbf{e}}}\left( \frac{r_1 \vec {n}_1\cdot \vec {n}_2}{2N_2}\right) \end{aligned}$$

and for $${\textbf{y}}\in ({{\mathbb {Z}}}/N{{\mathbb {Z}}})^g$$,

$$\begin{aligned} {\,{\textbf{e}}}_N(\vec {n}_2 \cdot {\textbf{y}})&= {\,{\textbf{e}}}\left( \frac{(N_2 r_2 + N_1 r_1) \vec {n}_2 \cdot {\textbf{y}}}{N_1N_2} \right) \\&= {\,{\textbf{e}}}\left( \frac{r_2 \vec {n}_2 \cdot {\textbf{y}}}{N_1} \right) \cdot {\,{\textbf{e}}}\left( \frac{r_1 \vec {n}_2 \cdot {\textbf{y}}}{N_2} \right) . \end{aligned}$$

By definition (1.2),

$$\begin{aligned} \begin{aligned} \{\operatorname {T}_N(\vec {n})\iota ^*(\varphi _1\otimes \varphi _2)\} ({\textbf{y}}) = {\,{\textbf{e}}}_{2N}(\vec {n}_1\cdot \vec {n}_2) {\,{\textbf{e}}}_N(\vec {n}_2 \cdot {\textbf{y}}) \iota ^*(\varphi _1\otimes \varphi _2) ({\textbf{y}}+\vec {n}_1) \end{aligned} \end{aligned}$$

so that, using (2.3), we obtain

$$\begin{aligned} \begin{aligned} \{\operatorname {T}_N(\vec {n})\iota ^*(\varphi _1\otimes \varphi _2)\} ({\textbf{y}})&= {\,{\textbf{e}}}_{2N}(\vec {n}_1\cdot \vec {n}_2) {\,{\textbf{e}}}_N(\vec {n}_2 \cdot {\textbf{y}}) \iota ^*(\varphi _1\otimes \varphi _2) ({\textbf{y}}+\vec {n}_1) \\&= {\,{\textbf{e}}}\left( \frac{r_2 \vec {n}_1\cdot \vec {n}_2}{2N_1}\right) e\left( \frac{r_2 \vec {n}_2 \cdot {\textbf{y}}}{N_1} \right) \varphi _1({\textbf{y}}+\vec {n}_1) \\&\quad \cdot {\,{\textbf{e}}}\left( \frac{r_1 \vec {n}_1\cdot \vec {n}_2}{2N_2}\right) {\,{\textbf{e}}}\left( \frac{r_1 \vec {n}_2 \cdot {\textbf{y}}}{N_2} \right) \varphi _2({\textbf{y}}+\vec {n}_1)\\&= \left( \operatorname {T}_{N_1}^{(r_2)}(\vec {n})\varphi _1 \right) ({\textbf{y}}) \cdot \left( \operatorname {T}_{N_2}^{(r_1)}(\vec {n})\varphi _2 \right) ({\textbf{y}}) \\&=\iota ^*\left( \left( \operatorname {T}_{N_1}^{(r_2)}(\vec {n}) \otimes \operatorname {T}_{N_2}^{(r_1)}(\vec {n}) \right) \left( \varphi _1\otimes \varphi _2 \right) \right) ({\textbf{y}}) \end{aligned} \end{aligned}$$

as claimed. $$\square $$

### Factorization of the quantized map

We continue to assume a factorization of $N=N_1 \cdot N_2$ with $N_1>1$, $N_2>1$ coprime, and such that $r_1,r_2$ satisfy (2.4). Then the Chinese Remainder Theorem induces an isometry

$$\begin{aligned} {{{\mathcal {H}}}}_{N}\simeq {\mathcal {H}}_{N_1} \otimes {\mathcal {H}}_{N_2}, \end{aligned}$$

which is respected by the translation operators (see Lemma 2.1). Furthermore, from now on, we identify the spaces ${{{\mathcal {H}}}}_{N}$ and ${\mathcal {H}}_{N_1} \otimes {\mathcal {H}}_{N_2}$ and thus we do not use the isomorphism map $\iota ^*$ anymore. We argue that we get a corresponding factorization of the quantized map $U_N(A) = U_{N,1}(A)$ defined by (1.5) as a tensor product:

#### [Style2 Style2 Style2]Lemma 2.2

There is some $\zeta \in {{\mathbb {C}}}$, with $|\zeta |=1$, such that we have a factorization

$$\begin{aligned} U_{N}(A) = \zeta U_{N_1,r_2}(A) \otimes U_{N_2,r_1}(A). \end{aligned}$$

#### Proof

We saw in Lemma 2.1 that

$$\begin{aligned} \operatorname {T}_N(\vec {n}A) = \operatorname {T}_{N_1}^{(r_2)}(\vec {n}A) \otimes \operatorname {T}_{N_2}^{(r_1)}(\vec {n}A). \end{aligned}$$

By (2.2), we have

$$\begin{aligned} \operatorname {T}_{N_1}^{(r_2)}(\vec {n}A) = U_{N_1,r_2}(A)^* \operatorname {T}_{N_1}^{(r_2)}(\vec {n}) U_{N_1,r_2}(A) \end{aligned}$$

and

$$\begin{aligned} \operatorname {T}_{N_2}^{(r_1)}(\vec {n}A) = U_{N_2,r_1}(A)^* \operatorname {T}_{N_2}^{(r_1)}(\vec {n}) U_{N_2,r_1}(A). \end{aligned}$$

Hence $${{\widetilde{U}}}= U_{N_1,r_2}(A)\otimes U_{N_2,r_1}(A)$$ satisfies

$$\begin{aligned} \begin{aligned} {{\widetilde{U}}}^* \operatorname {T}_N(\vec {n}) {{\widetilde{U}}}&= {{\widetilde{U}}}^* \left( \operatorname {T}_{N_1}^{(r_2)}(\vec {n}) \otimes \operatorname {T}_{N_2}^{(r_1)}(\vec {n}) \right) {{\widetilde{U}}} \\&= \operatorname {T}_{N_1}^{(r_2)}(\vec {n}A)\otimes \operatorname {T}_{N_2}^{(r_1)}(\vec {n}A)=\operatorname {T}_N(\vec {n}A) \end{aligned} \end{aligned}$$

for all $\vec {n}\in {{\mathbb {Z}}}^{2g}$. Since $U_N(A)$ is the unique (up to a scalar multiple) unitary operator satisfying this relation, we must have that ${{\widetilde{U}}}$ is a scalar multiple, of absolute value one, of $U_N(A)$. $\square$

## Bounding $\operatorname {T}_N$ via the Tensor Product Structure

### Basic properties of tensor products

We first recall a useful identity regarding operator norms of tensor products. Let *V*, *W* be finite dimensional inner product spaces, let $\operatorname {T}_V:~V \rightarrow V$ and $\operatorname {T}_W:~W \rightarrow W$ be linear maps, and let $\Vert \operatorname {T}_V\Vert$ and $\Vert \operatorname {T}_W\Vert$ denote the operator norms of $\operatorname {T}_V$ and $\operatorname {T}_W$, respectively. There is a natural inner product on the tensor product $V \otimes W$ — given orthonormal bases $\{v_{1},\ldots ,v_{n}\}$ and $\{w_{1},\ldots , w_{m}\}$ for *V*, *W*, respectively, declare $\{ v_{i} \otimes w_{j}\}_{1 \leqslant i \leqslant n, 1 \leqslant j \leqslant m}$ to be an orthonormal basis for $V \otimes W$. We then have the relation

3.1 $$\begin{aligned} \Vert \operatorname {T}_V \otimes \operatorname {T}_W\Vert = \Vert \operatorname {T}_V\Vert \cdot \Vert \operatorname {T}_W\Vert , \end{aligned}$$

between the operator norms (cf. [21, Page 299, Proposition]).

### Eigenspace decomposition in the coprime case

Assume that $\operatorname {T}_V$ and $\operatorname {T}_W$ are both diagonalizable, with eigenvalues being roots of unity (in particular there exists, say minimal, integers $t_{1},t_{2} > 0$ such that $\operatorname {T}_V^{t_{1}} = I_V$ and $\operatorname {T}_W^{t_{2}} = I_W$, where $I_V$ and $I_W$ are the corresponding identity operators; note that we do not assume that $\operatorname {T}_V$ and $\operatorname {T}_W$ have the same dimensions).

The eigenspaces of $\operatorname {T}_V \otimes \operatorname {T}_W$ are particularly easy to describe in terms of the eigenspaces of $\operatorname {T}_V$ and $\operatorname {T}_W$ when $\gcd (t_{1},t_{2})=1$. Namely, let $\{V_{i} \}_{i}$ denote the eigenspaces of $\operatorname {T}_V$, and let $\{W_{j}\}_{j}$ denote the eigenspaces of $\operatorname {T}_W$; here we allow both $\operatorname {T}_V,\operatorname {T}_W$ to have eigenvalues with multiplicities. The eigenspaces of $\operatorname {T}_V \otimes \operatorname {T}_W$ are then given by $\{ V_{i} \otimes W_{j} \}_{i,j}$. Further, if the eigenvalue associated with $V_{i}$ is denoted $\mu _{i}$ and the eigenvalue associated with $W_{j}$ is denoted by $\nu _{j}$, all eigenvalues of $\operatorname {T}_V \otimes \operatorname {T}_W$ are of the form $\lambda _{i,j} = \mu _{i} \nu _{j}$. In particular, if $\gcd (t_{1},t_{2})=1$ we find that $\mu _{i_{1}} \nu _{j_{1}} = \mu _{i_{2}} \nu _{j_{2}}$ implies that $i_{1}=i_{2}$ and $j_{1}=j_{2}$. Informally, all multiplicities arise by combining multiplicities from the $V_{i},W_{j}$ eigenspaces (note that this is not true if $\gcd (t_{1},t_{2})>1$).

### Bounds using the tensor product structure

We now bound matrix coefficients of the special form $\langle \operatorname {T}_N(\vec {n}) \psi , \psi ' \rangle$, where, as in § 1.3,

$$\begin{aligned}\operatorname {T}_N(\vec {n})= \operatorname {Op}_N({\,{\textbf{e}}}\left( {\textbf{x}}\cdot \vec {n}\right) ) \end{aligned}$$

and $\psi ,\psi '$ are eigenfunctions of $U_{N}(A)$.

Let *B* be an element of $\operatorname {Sp}(2g,{{\mathbb {Z}}})$. Assume that $N= N_1N_2$ with coprime integers $N_1>1$, $N_2>1$. Let $t_{i}$ denote the order of $B \,\operatorname {mod}{N_i}$, $i=1,2$. Further assume that

3.2 $$\begin{aligned} \gcd (t_{1},t_{2})=1. \end{aligned}$$

Let $r_1,r_2\in {{\mathbb {Z}}}$ satisfy (2.4). Taking $A=B$ in Lemmas 2.1 and 2.2, we find that

$$\begin{aligned} \operatorname {T}_N(\vec {n})= \operatorname {T}_{N_1}^{(r_2)}(\vec {n}) \otimes \operatorname {T}_{N_2}^{(r_1)}(\vec {n})\quad \text {and}\quad U_{N}(B) = \zeta U_{N_1,r_{2}}(B)\otimes U_{N_2,r_{1}}(B) \end{aligned}$$

for some $\zeta \in {{\mathbb {C}}}^*$ with $|\zeta |=1$.

#### [Style2 Style2 Style2]Lemma 3.1

Let $\psi , \psi '$ denote norm one eigenfunctions of $U_{N}(B)$. With assumptions as above, we then have

$$\begin{aligned} |\langle \operatorname {T}_N(\vec {n})\psi , \psi ' \rangle | \leqslant \max _{\varphi , \varphi ' \in \Phi _{N_1, r_2}} | \langle \operatorname {T}_{N_1}^{(r_2)} (\vec {n})\varphi , \varphi ' \rangle |, \end{aligned}$$

where $\Phi _{N_1, r_2}$ denotes the set of all eigenfunctions of $U_{N_1,r_{2}}(B)$ of norm one.

#### Proof

Let $E,E'$ denote the eigenspaces of $U_{N}(B)$ containing $\psi , \psi '$, and let $\lambda , \lambda '$ denote the corresponding eigenvalues.

By the discussion in § 3.2, we have

3.3 $$\begin{aligned} E = V_1 \otimes V_2, \end{aligned}$$

where $$V_1$$ and $$V_2$$ are eigenspaces of $$U_{N_1,r_{2}}(B)$$ and $$ U_{N_2,r_{1}}(B)$$, respectively, and similarly we have $$E' = V_{1}' \otimes V_{2}'$$. Thus, if we let $$S = P_{E'} \operatorname {T}_N(\vec {n}) P_{E}$$, with $$P_{E}: {{{\mathcal {H}}}}_{N}\rightarrow E$$ denoting the orthogonal projection onto *E* (and similarly for $$P_{E'}$$), we have

3.4 $$\begin{aligned} \max _{\begin{array}{c} \psi \in E, \, \psi ' \in E', \\ \Vert \psi \Vert =\Vert \psi '\Vert =1 \end{array}} |\langle \operatorname {T}_N(\vec {n}) \psi , \psi ' \rangle | = \Vert S\Vert . \end{aligned}$$

Now, the decomposition (3.3) and Lemma 2.1, after a simple calculation, give that

3.5 $$\begin{aligned} S = S_{1} \otimes S_{2} \end{aligned}$$

where $S_{1} = P_{V_1'} \operatorname {T}_{N_1}^{(r_2)}(\vec {n}) P_{V_1}$ and $S_{2} = P_{V_2'} \operatorname {T}_{N_2}^{(r_1)}(\vec {n}) P_{V_2}$. Since both $S_{1},S_{2}$ arise as compositions of the unitary maps $\operatorname {T}_{N_1}^{(r_2)}(\vec {n}),\operatorname {T}_{N_2}^{(r_1)}(\vec {n})$ with orthogonal projections, they are both sub-unitary, and we have the trivial bounds $\Vert S_{1}\Vert ,\Vert S_{2}\Vert \leqslant 1$. Thus, by the operator norm identity of tensor products (3.1), we see from (3.5) that

$$\begin{aligned}\Vert S\Vert = \Vert S_{1}\Vert \cdot \Vert S_{2}\Vert \leqslant \Vert S_{1}\Vert . \end{aligned}$$

Using this, together with

$$\begin{aligned} \Vert S_{1}\Vert = \max _{\begin{array}{c} \varphi \in V_1, \varphi ' \in V_1' \\ \Vert \varphi \Vert =\Vert \varphi '\Vert =1 \end{array}} | \langle \operatorname {T}_{N_1}^{(r_2)}(\vec {n}) \varphi , \varphi ' \rangle |, \end{aligned}$$

and recalling (3.4), the result now follows. $\square$

We now consider a more general case when instead of the coprimality condition (3.2) we have

$$\begin{aligned} \gcd (t_{1},t_{2})=d. \end{aligned}$$

Since any eigenfunction of $U_{N}(A)$ is an eigenfunction for $U_{N}\left( A^d\right)$ for any integer $d> 0$, we have

3.6 $$\begin{aligned} \max _{\psi , \psi ' \in \Psi _N} | \langle \operatorname {T}_N(\vec {n}) \psi , \psi ' \rangle | \leqslant \max _{{{\widetilde{\psi }}}, {{\widetilde{\psi }}}' \in {{\widetilde{\Psi }}}_{N,d}} | \langle \operatorname {T}_N(\vec {n}) {{\widetilde{\psi }}}, {{\widetilde{\psi }}}'\rangle |, \end{aligned}$$

where $\Psi _N$ and ${{\widetilde{\Psi }}}_{N,d}$ denote the set of normalized eigenfunctions of $U_{N}(A)$ and $U_{N}(A^{d})$, respectively.

## Congruences and Exponential Sums

### Reduction to a counting problem

For a (row) vector $\vec {n}\in {{\mathbb {Z}}}^{2g}$, $\vec {n}\ne {{\textbf{0}}}\bmod N$, we denote by $Q_{2\nu }(N;\vec {n})$ the number of solutions of the congruence

4.1 $$\begin{aligned} \vec {n}\left( A^{k_1}+\ldots +A^{k_{2\nu }} -A^{\ell _1} -\ldots -A^{\ell _{2\nu }}\right) \equiv {{\textbf{0}}}\bmod N, \end{aligned}$$

with $$1\leqslant k_i,\ell _i \leqslant \operatorname {ord}(A,N) $$, $$i =1, \ldots , 2\nu $$.

The key inequality below connects the $4\nu$-th moment associated to the basic observables $\operatorname {T}_N^{(r)}(\vec {n})$ with the number of solutions to the system (4.1). This kind of inequality (for $\nu =1$) underlies the argument of [19], and also the argument of [2].

#### [Style2 Style2 Style2]Lemma 4.1

Let ${{\textbf{0}}}\ne \vec {n}\in {{\mathbb {Z}}}^{2g}$ and let *r* be an integer coprime to *N*. Then

4.2 $$\begin{aligned} \max _{\psi ,\psi '} \left| \langle \operatorname {T}_N^{(r)}(\vec {n})\psi ,\psi ' \rangle \right| ^{4\nu } \leqslant N^g \frac{Q_{2\nu }(N;\vec {n})}{\operatorname {ord}(A,N)^{4\nu } }, \end{aligned}$$

where the maximum is taken over all pairs of normalized eigenfunctions of $U_{N,r}(A)$.

#### Proof

We abbreviate $\tau =\operatorname {ord}(A,N)$. Given a pair $(\psi ,\psi ')$ of normalized eigenfunctions of $U_{N,r}(A)$ with eigenvalues $\lambda ,\lambda '$, put $\mu = \lambda '/\lambda$ and note that $\mu$ is a root of unity (since $\lambda$ and $\lambda '$ are). Let

$$\begin{aligned} D(\vec {n}) = \frac{1}{\tau }\sum _{i=1}^\tau U_{N,r}(A)^{-i} \operatorname {T}_N^{(r)}(\vec {n}) U_{N,r}(A)^i \mu ^{i} = \frac{1}{\tau }\sum _{i=1}^\tau \operatorname {T}_N^{(r)}(\vec {n}A^i) \mu ^{i} \end{aligned}$$

be the $\mu$-twisted time averaged observable, where the last equality comes from (2.2). Then for any pair of eigenfunctions $(\psi , \psi ')$ of $U_{N,r}(A)$, with eigenvalues $\lambda$ and $\lambda '$, we have

$$\begin{aligned} \langle \operatorname {T}_N^{(r)}(\vec {n})\psi ,\psi ' \rangle =\langle D(\vec {n})\psi ,\psi ' \rangle , \end{aligned}$$

see also the proof of [19, Proposition 4]. Put $H(\vec {n}) = D(\vec {n})^{*}D(\vec {n})$; note that $H(\vec {n})$ is Hermitian. Clearly

$$\begin{aligned} |\langle D(\vec {n})\psi ,\psi ' \rangle | \leqslant \Vert D(\vec {n}) \Vert = \Vert H(\vec {n}) \Vert ^{1/2}, \end{aligned}$$

where $\Vert H(\vec {n}) \Vert$ denotes the operator norm of $H(\vec {n})$. Therefore for any $\nu \ge 1$,

$$\begin{aligned} | \langle \operatorname {T}_N^{(r)}(\vec {n})\psi ,\psi ' \rangle |^{4\nu } \leqslant \Vert H(\vec {n})\Vert ^{2\nu } = \Vert H(\vec {n})^{\nu }\Vert ^{2}. \end{aligned}$$

We bound the operator norm by the *Hilbert–Schmidt norm* and obtain

$$\begin{aligned} \Vert H(\vec {n})^{\nu }\Vert ^{2}\leqslant \Vert H(\vec {n})^{\nu }\Vert _{HS}^{2} = \operatorname {tr}((H(\vec {n})^{\nu })^{*}H(\vec {n})^{\nu }) = \operatorname {tr}(H(\vec {n})^{2\nu }), \end{aligned}$$

where $\operatorname {tr}$ denotes the operator trace. Finally, compute

$$\begin{aligned} \begin{aligned} H(\vec {n})^{2\nu }&= \frac{1}{\tau ^{4\nu }} \sum _{k_1,\ldots ,k_{2\nu },\ell _1\ldots ,\ell _{2\nu }=1}^\tau \\&\quad \times \prod _{j=1}^{2\nu } \left( \operatorname {T}_N^{(r)}\left( \vec {n}A^{k_j}\right) \operatorname {T}_N^{(r)}\left( -\vec {n}A^{\ell _j}\right) \right) \mu ^{ \sum _{j=1}^{2\nu }(k_j-\ell _j)} \\&= \frac{1}{\tau ^{4\nu }} \sum _{k_1,\ldots ,k_{2\nu },\ell _1\ldots ,\ell _{2\nu }=1}^\tau \gamma ({\textbf{k}},\varvec{\ell },\mu  ) \operatorname {T}_N^{(r)}\left( \vec {n}\sum _{j=1}^{2\nu } \left( A^{k_j}- A^{\ell _j} \right) \right) \end{aligned} \end{aligned}$$

with some complex coefficients $\gamma ({\textbf{k}}, \varvec{\ell }, \mu )$, satisfying $|\gamma ({\textbf{k}}, \varvec{\ell }, \mu )|=1$, where ${\textbf{k}}= \left( k_1,\ldots ,k_{2\nu }\right)$ and $\varvec{\ell }= \left( \ell _1\ldots ,\ell _{2\nu }\right)$, and where the last equality comes from (2.1). Taking the trace and using

$$\begin{aligned} |\operatorname {tr}\operatorname {T}_N^{(r)}({\textbf{m}})| = {\left\{ \begin{array}{ll} N^g &  \text {if }\textbf{m}={{\textbf{0}}}\bmod N, \\ 0&  \text {otherwise,} \end{array}\right. } \end{aligned}$$

we find

$$\begin{aligned} \left| \langle \operatorname {T}_N^{(r)}(\vec {n})\psi ,\psi ' \rangle \right| ^{4\nu } \leqslant \operatorname {tr}\left( H(\vec {n})^{2\nu }\right) \leqslant \frac{N^g}{\tau ^{4\nu }} Q_{2\nu }(N;\vec {n}) \end{aligned}$$

which concludes the proof. $\square$

#### Remark 4.2

If the right hand side of (4.2) tends to zero, then (1.7) is satisfied, that is, all eigenfunctions of $U_N(A)$ are uniformly distributed, and more generally, all off-diagonal matrix coefficients tend to zero. Thus Lemma 4.1 reduces the problem (1.7) to a purely arithmetic issue.

### Linear independence of matrix powers

The primal goal of this section is to show that if the characteristic polynomial $f_A$ of *A* is separable over ${{\mathbb {Q}}}$ we can essentially eliminate the dependence on the vector $\vec {n}$ in our argument, except in some special cases. In particular, instead of $Q_{2\nu }(N;\vec {n})$ we can consider the number of solutions of the congruence

4.3 $$\begin{aligned} A^{k_1}+\ldots +A^{k_{2\nu }} \equiv A^{\ell _1} +\ldots +A^{\ell _{2\nu }}\bmod N, \end{aligned}$$

with $1\leqslant k_i,\ell _i \leqslant \operatorname {ord}(A,N)$, $i =1, \ldots , 2\nu$. This is based on the following result which is also used in our bounds on exponential sums. However, we first need to introduce the notion of zero-divisors amongst the row vectors $\vec {n}\in {{\mathbb {Z}}}^{2g}$. For this we first identify ${{\mathbb {Q}}}^{2g}\cong {{\mathbb {Q}}}[X]/(f_A(X))$ as a ${{\mathbb {Q}}}[A]$ module. We say that $\vec {n}$ is a *zero-divisor*, if its image ${{\widetilde{\vec {n}}}} \in {{\mathbb {Q}}}[X]/(f_A(X))$ is a zero-divisor in this module (we follow the convention that zero is also a zero divisor, call all other zero divisors *nontrivial.*)

#### [Style2 Style2 Style2]Lemma 4.3

Let $A\in \operatorname {Sp}(2g,{{\mathbb {Z}}})$ have a separable characteristic polynomial. Then for any row vector $\vec {n}\in {{\mathbb {Z}}}^{2g}$, which is not a zero-divisor, we have:

(i) the vectors $\vec {n}, \vec {n}A, \ldots ,\vec {n}A^{2g-1}$ are linearly independent;
(ii) there exists some $p_0(A)$, depending only on *A*, such that for all primes $p>p_0(A) \Vert \vec {n}\Vert _2^{2g}$, the vectors $\vec {n}, \vec {n}A, \ldots ,\vec {n}A^{2g-1}$ are linearly independent modulo *p*.

#### Proof

In the case when the characteristic polynomial is irreducible, Part (i) is proved in [19, page 210] (for $n=2$) and in the proof of [20, Theorem 2.5] (which is done over a finite field but it remains valid over any field).

In our more general case of separability, assume that the vectors $\vec {n}A^{i}$, $i =0, \ldots , 2g-1$, are linearly dependent over ${{\mathbb {Q}}}$, that is, there is a linear relation

$$\begin{aligned} \sum _{i=0}^{2g-1} c_i \vec {n}A^{i}=\vec {n}\left( \sum _{i=0}^{2g-1} c_i A^{i}\right) = {\textbf{0}} \end{aligned}$$

for some $c_i\in {{\mathbb {Q}}}$ not all zero. Since $\vec {n}$ is not a zero-divisor, we obtain

$$\begin{aligned} \sum _{i=0}^{2g-1} c_i A^{i} = {\textbf{0}}. \end{aligned}$$

However, this shows that the minimal polynomial of *A* has degree at most $2g-1$, which contradicts the fact that the minimal polynomial is $f_A$ since it is separable. This concludes the proof of Part (i).

To show Part (ii) one considers the determinant of the matrix having rows $\textbf{n}, \ldots ,\textbf{n} A^{2g-1}$ whose vanishing is equivalent to linear independence; it is an integer, nonzero by Part (i), hence for all primes *p* not dividing it we have linear independence mod *p*. $\square$

### Reduction to a system of exponential equations

We now consider (4.1) for a prime $N=p$.

Let $A\in \operatorname {Sp}(2g,{{\mathbb {Z}}})$ have separable characteristic polynomial $f_A\in {{\mathbb {Z}}}[X]$. Assume that *p* is large enough so that $f_A$ is separable modulo *p*, which also implies that *A* is diagonalisable over $\overline{{{\mathbb {F}}}}_{p}$.

Next, let

$$\begin{aligned} f_A(X)= h_1(X)\cdots h_t(X) \bmod p \end{aligned}$$

be the factorization of $f_A$ into irreducible factors $h_i\in {{\mathbb {F}}}_p[X]$ of degrees $d_i=\deg h_i$, $i=1\ldots , t$. In particular, any root of $h_i$ belongs to ${{\mathbb {F}}}_{p^{d_i}}$, $i=1\ldots , t$. For each $h_i$ we fix a root $\lambda _i \in {{\mathbb {F}}}_{p^{d_i}}$ and consider the system of equations (in the algebraic closure of ${{\mathbb {F}}}_p$)

4.4 $$\begin{aligned} \lambda _i^{k_1}+\cdots +\lambda _i^{k_{2\nu }}= \lambda _i^{\ell _1}+\cdots +\lambda _i^{\ell _{2\nu }}, \qquad i=1\ldots , t, \end{aligned}$$

with $1\leqslant k_j,\ell _j \leqslant \operatorname {ord}(A,p)$, $j= 1,\ldots , 2\nu$. It is easy to see that for all choices of roots $\lambda _1, \ldots , \lambda _t$ we get equivalent systems.

Next, we reduce counting the number of solutions to (4.3) to counting the number of solutions to (4.4).

In fact our treatment depends only on the degrees $d_1, \ldots , d_t$ and so we denote the number of solutions to (4.4) by $R_{2\nu }(d_1,\ldots ,d_t;p)$.

#### [Style2 Style2 Style2]Lemma 4.4

Under the above assumptions, there exists some $p_0(A)$, depending only on *A*, such that for any vector $\vec {n}\in {{\mathbb {Z}}}^{2g}$, which is not a zero divisor, and $p > p_0(A) \Vert \vec {n}\Vert _2^{2g}$ we have

$$\begin{aligned} Q_{2\nu }(p;\vec {n}) = R_{2\nu }(d_1,\ldots ,d_t;p). \end{aligned}$$

#### Proof

Let us denote

$$\begin{aligned} B=A^{k_1}+\dots +A^{k_{2\nu }} -A^{\ell _1} -\dots -A^{\ell _{2\nu }}. \end{aligned}$$

Multiplying (4.1) by powers of *A*, we conclude that

$$\begin{aligned} \left( \vec {n}A^i\right) B \equiv {{\textbf{0}}}\bmod p,\qquad i=0,\ldots ,2g-1, \end{aligned}$$

which is equivalent to

$$\begin{aligned} \begin{pmatrix} \vec {n}\\ \vec {n}A\\ \cdots \\ \vec {n}A^{2g-1} \end{pmatrix}B \equiv {{\textbf{0}}}\bmod p. \end{aligned}$$

From Lemma 4.3 (ii), there exists some $p_0(A)$, depending only on *A*, such that for $p > p_0(A) \Vert \vec {n}\Vert _2^{2g}$ all rows $\vec {n}, \vec {n}A, \ldots ,\vec {n}A^{2g-1}$ are linearly independent modulo *p*, and thus from the above we conclude that *B* vanishes over ${{\mathbb {F}}}_p$.

Since *A* is diagonalisable over $\overline{{{\mathbb {F}}}}_{p}$, the equation above is equivalent to

$$\begin{aligned} \Lambda ^{k_1}+\ldots +\Lambda ^{k_{2\nu }} \equiv \Lambda ^{\ell _1} + \ldots +\Lambda ^{\ell _{2\nu }} \bmod p, \end{aligned}$$

where $\Lambda$ is a diagonal matrix with elements on the diagonal all the roots of $h_i$, $i=1,\ldots , t$. Since for each irreducible factor of *f* modulo *p*, all roots are conjugate (that is, the roots of $h_i$ in ${{\mathbb {F}}}_{p^{d_i}}$ are $\lambda _i,\lambda _i^p,\ldots ,\lambda _i^{p^{d_i-1}}$), we conclude the proof. $\square$

## Multiplicative Orders and Exponential Sums

### Ergodicity and the order modulo *p*

It is natural that our argument, as in [2, 19, 20], rests on various results on multiplicative orders.

We begin by showing that the multiplicative orders of the eigenvalues of $A\in \operatorname {Sp}(2g,{{\mathbb {Z}}})$, and their ratios, are sufficiently large for almost all primes. The argument is a modification of that of Hooley [13].

We recall the definition of $\operatorname {ord}(\lambda ,p)$ in § 1.4 and also that we say that *p* is split prime if the characteristic polynomial of the matrix *A* splits completely modulo *p*.

#### [Style2 Style2 Style2]Lemma 5.1

Assume that $A\in \operatorname {Sp}(2g,{{\mathbb {Z}}})$ has separable characteristic polynomial and that no eigenvalue or ratio of distinct eigenvalues is a root of unity. Let $\lambda _1,\ldots ,\lambda _{2g}$ be the eigenvalues of *A*. Then for almost all split primes *p* we have

$$\begin{aligned} \operatorname {ord}(\lambda _i,p), \operatorname {ord}(\lambda _i/\lambda _j,p) > p^{1/2}/\log p, \quad 1\leqslant i\ne j \leqslant 2g. \end{aligned}$$

#### Proof

For a sufficiently large $Y\geqslant 2$, let

$$\begin{aligned} A(Y) = \prod _{n\leqslant Y} \prod _{1 \leqslant i \leqslant 2g} {\textrm{Nm}}_{K/{{\mathbb {Q}}}}(\lambda _i^n - 1) \prod _{1 \leqslant j < h \leqslant 2g} {\textrm{Nm}}_{K/{{\mathbb {Q}}}}(\lambda _j^n - \lambda _h^n), \end{aligned}$$

where ${\textrm{Nm}}_{K/{{\mathbb {Q}}}}\left( \zeta \right)$ is the *norm* of $\zeta \in K= Q\left( \lambda _1, \ldots , \lambda _n\right)$ in ${{\mathbb {Q}}}$. Note that $A(Y) \ne 0$ because of the condition on the avoidance of roots of unity among the eigenvalues and their ratios, and $A(Y) \in {{\mathbb {Z}}}$ since all eigenvalues are algebraic integers. Since

$$\begin{aligned} {\textrm{Nm}}_{K/{{\mathbb {Q}}}}\left( \lambda _i^n - 1\right) =\prod _{\sigma \in \textrm{Gal}(K/{{\mathbb {Q}}})}\left( \sigma (\lambda _i)^n - 1\right) \end{aligned}$$

and

$$\begin{aligned} {\textrm{Nm}}_{K/{{\mathbb {Q}}}}\left( \lambda _j^n - \lambda _h^n\right) =\prod _{\sigma \in \textrm{Gal}(K/{{\mathbb {Q}}})}\left( \sigma (\lambda _j)^n-\sigma (\lambda _h)^n\right) , \end{aligned}$$

where both products are over all automorphisms $\sigma$ from the Galois group $\textrm{Gal}(K/{{\mathbb {Q}}})$ of *K* over ${{\mathbb {Q}}}$, and thus

$$\begin{aligned} \log {\textrm{Nm}}_{K/{{\mathbb {Q}}}}(\lambda _i^n - 1), \, \log {\textrm{Nm}}_{K/{{\mathbb {Q}}}}(\lambda _j^n - \lambda _h^n)\ll n, \end{aligned}$$

we see that

5.1 $$\begin{aligned} \log |A(Y)| \ll Y^2. \end{aligned}$$

Let ${{\mathcal {P}}}(Y)$ be the set of primes for which

$$\begin{aligned} \min _{1 \leqslant i \leqslant 2g} \min _{1 \leqslant j < h \leqslant 2g} \{\operatorname {ord}(\lambda _i,p), \operatorname {ord}(\lambda _j/\lambda _h,p)\} \leqslant Y. \end{aligned}$$

We observe that for $p\in {{\mathcal {P}}}(Y)$, we must have $p\mid A(Y)$, and hence

5.2 $$\begin{aligned} \sharp {{\mathcal {P}}}(Y) \leqslant \omega \left( A(Y)\right) , \end{aligned}$$

where, as usual, $\omega (k)$ denotes the number of prime divisors of the integer $k \geqslant 1$. From the trivial observation that $\omega (k)! \leqslant k$ and the Stirling formula, we derive

5.3 $$\begin{aligned} \omega (k) \ll \frac{\log k }{\log \log (k+2)}, \qquad k \geqslant 1. \end{aligned}$$

Putting together (5.1), (5.2) and (5.3), we see that

$$\begin{aligned} \sharp {\mathcal {P}}(Y)\ll Y^2 /\log Y. \end{aligned}$$

Since the number of primes $p\leqslant X$ is $\pi (X) \sim X/\log X$, we can take $Y=\sqrt{X}/\log X$ to assure that for all but $o\left( \pi (X)\right)$ primes $p \leqslant X$, we have

$$\begin{aligned} \operatorname {ord}(\lambda _i,p), \operatorname {ord}(\lambda _i/\lambda _j,p) >\sqrt{X}/\log X\ge \sqrt{p}/\log p,\quad 1 \leqslant i \ne j \leqslant 2g. \end{aligned}$$

Since splitting fields of polynomials are Galois extensions, by the Chebotarev Density Theorem, see [14, Theorem 21.2], for a positive proportion of primes *p*, see our convention in § 1.4, the characteristic polynomial of the matrix *A* splits modulo *p*. This concludes the proof. $\square$

### Relation with short exponential sums

As discussed in § 4.1, one relates the uniform distribution of the eigenfunctions of the operator $U_N(A)$, as well as the decay of off-diagonal matrix elements, to bounding the number of solutions $Q_{2\nu }(N;\textbf{n})$ for $\textbf{n}\in {{\mathbb {Z}}}^{2g}$ to the matrix congruence (4.1), see Lemma 4.1.

Following the discussion after Theorem 1.2 and Lemma 4.1, we thus reduce the problem to showing that

$$\begin{aligned} Q_{2\nu }(p;\textbf{n}) = o\left( \frac{\operatorname {ord}(A,p)^{4\nu }}{p^g} \right) \end{aligned}$$

for a set of ‘good’ primes *p* for which the characteristic polynomial of *A* splits completely over ${{\mathbb {F}}}_p$, with eigenvalues $\lambda _i\in {{\mathbb {F}}}_p^*$, $i=1,\ldots ,2g$.

In turn, using the orthogonality of exponential sums, this leads us to a problem of obtaining nontrivial cancellation in exponential sums of the form

$$\begin{aligned} \sum _{j=1}^{\operatorname {ord}(A,p)} {\mathbf {\,e}}_p\left( \alpha _1 \lambda _1^{j} + \ldots + \alpha _{2g}\lambda _{2g}^{j}\right) \end{aligned}$$

for $$(\alpha _1,\ldots ,\alpha _{2g})\in {{\mathbb {F}}}_p^{2g}$$.

These exponential sums are not treatable by algebro-geometric methods of Weil and Deligne, but fortunately they can be treated by methods from additive combinatorics. In particular, we make use of the bounds of Bourgain [1, Corollary] on Mordell type sums over prime fields.

#### [Style2 Style2 Style2]Lemma 5.2

For every $\varepsilon > 0$ there exists some $\delta > 0$ such that the following holds. Let $\alpha _1, \ldots , \alpha _s \in {{\mathbb {F}}}_p$, not all zero, and $\lambda _1,\ldots , \lambda _s\in {{\mathbb {F}}}_p^*$ be such that

$$\begin{aligned} \operatorname {ord}(\lambda _i,p),\quad \operatorname {ord}(\lambda _i/\lambda _j,p) \geqslant p^{\varepsilon }, \qquad 1\leqslant i, j\leqslant s, \ i \ne j. \end{aligned}$$

Then

$$\begin{aligned} \left| \sum _{x=1}^{T} {\mathbf {\,e}}_p\left( \alpha _1 \lambda _1^{x} + \ldots + \alpha _{s}\lambda _s^{x}\right) \right| \ll T p^{-\delta }, \end{aligned}$$

where *T* is the order of the subgroup of ${{\mathbb {F}}}_p^*$ generated by $\lambda _1,\ldots , \lambda _s$.

According to Lemma 4.4, in the split case, that is, for $\lambda _i\in {{\mathbb {F}}}_p^*$, $i=1,\ldots ,2g$, the number of solutions to the system (4.4) is given by $Q_{2\nu }(p;\textbf{n})=R_{2\nu }(1,\ldots ,1;p)$ (with $t=2g$ therein). Using the orthogonality of exponential functions we obtain

$$\begin{aligned} \begin{aligned} Q_{2\nu }(p;\textbf{n})&=R_{2\nu }(1,\ldots ,1;p) \\&= \frac{1}{p^{2g}} \sum _{\mathbf {\alpha }\in {{\mathbb {F}}}_p^{2g}} \left| \sum _{t=1}^T {\mathbf {\,e}}_p\left( \alpha _1 \lambda _1^t+\ldots +\alpha _{2g}\lambda _{2g}^t\right) \right| ^{4\nu } \\&< \frac{T^{4\nu }}{p^{2g}} + \max _{{{\textbf{0}}}\ne \mathbf {\alpha }\in {{\mathbb {F}}}_p^{2g}} \left| \sum _{t=1}^T {\mathbf {\,e}}_p\left( \alpha _1 \lambda _1^t+\ldots +\alpha _{2g}\lambda _{2g}^t\right) \right| ^{4\nu }. \end{aligned} \end{aligned}$$

Inserting Lemma 5.2, exactly as in [2], we derive

$$\begin{aligned} Q_{2\nu }(p;\textbf{n})\ll \frac{T^{4\nu }}{p^{2g}} + T^{4\nu } p^{-4\nu \delta } \leqslant 2 \frac{T^{4\nu }}{p^{2g}} \end{aligned}$$

for $\nu \ge g/(2\delta )$. Hence we find:

#### [Style2 Style2]Corollary 5.3

Let $A\in \operatorname {Sp}(2g,{{\mathbb {Z}}})$ have separable characteristic polynomial. For every $\varepsilon > 0$ there exists some integer $\nu _0 > 0$ such that the following holds. For a prime *p* so that *A* splits modulo *p*, let the eigenvalues of *A* be

$$\begin{aligned} \lambda _1,\ldots , \lambda _{2g}\in {{\mathbb {F}}}_p^*. \end{aligned}$$

Assume that

$$\begin{aligned} \operatorname {ord}(\lambda _i,p),\quad \operatorname {ord}(\lambda _i/\lambda _j,p) \geqslant p^{\varepsilon }, \qquad 1\leqslant i, j\leqslant 2g, \ i \ne j. \end{aligned}$$

Then, for any vector $\vec {n}\in {{\mathbb {Z}}}^{2g}$, which is not a zero-divisor and such that for $p > p_0(A) \Vert \vec {n}\Vert _2^{2g}$, where $p_0(A)$ is as in Lemma 4.4, and all $\nu \ge \nu _0$, we have

$$\begin{aligned} Q_{2\nu }(p;\textbf{n}) \ll \frac{\operatorname {ord}(A,p)^{4\nu }}{p^{2g}}. \end{aligned}$$

### Bounding $\langle \operatorname {T}_p^{(r)}(\vec {n})\psi ,\psi ' \rangle$ for a positive proportion of primes

We remark that the assumptions of Lemma 5.2 and Corollary 5.3 hold for a positive proportion of primes *p* (in fact, for a full density subset of the set of primes *p* for which the characteristic polynomial of *A* splits completely, see Lemma 7.3). Hence, combining Lemma 4.1 and Corollary 5.3, we obtain the desired estimate (1.8) on $\langle \operatorname {T}_p^{(r)}(\vec {n}) \psi ,\psi ' \rangle$ when $\vec {n}$ is not a zero-divisor.

#### [Style2 Style2]Corollary 5.4

Let $A\in \operatorname {Sp}(2g,{{\mathbb {Z}}})$ have separable characteristic polynomial. There exists some constant $\gamma >0$, depending only on *A*, such that for a positive proportion of primes *p* the following holds: For all integers *r* coprime to *p*, and for any $\vec {n}\in {{\mathbb {Z}}}^{2g}$, which is not a zero-divisor and with $p > p_0(A) \Vert \vec {n}\Vert _2^{2g}$, where $p_0(A)$ is as in Lemma 4.4,

$$\begin{aligned} \max _{\psi , \psi ' } \left| \langle \operatorname {T}_p^{(r)}(\vec {n})\psi ,\psi ' \rangle \right| \leqslant p^{-\gamma }, \end{aligned}$$

the maximum over all pairs of normalized eigenvectors of $U_{p,r}(A)$.

## Treatment of Zero-Divisors

### Preliminaries

We remark that if $f_A$ is irreducible then there are no nontrivial zero-divisors, and thus the results of § 4.1 allow us to complete the proof. However in the case when $f_A$ is separable but not irreducible we need additional considerations to treat vectors $\vec {n}\in {{\mathbb {Z}}}^{2g}$ which are zero-divisors, as defined in § 4.2. Thus this section is not needed if one is only interested in the case of matrices $A \in \operatorname {Sp}(2g,{{\mathbb {Z}}})$ with irreducible characteristic polynomials.

### Remarks on symplectic spaces

We next record some basic facts regarding symplectic vector spaces. Let *W* be a symplectic space, that is, a vector space with a non-degenerate alternating bilinear form, which we denote $\langle \cdot , \cdot \rangle$. We note that a subspace $U \subseteq V$ is symplectic, that is, the restriction of the symplectic form to *U* is non-degenerate, if and only if $U \cap U^{\perp } = \{{{\textbf{0}}}\}$.

#### [Style2 Style2 Style2]Lemma 6.1

Let $A \in \operatorname {Sp}(V)$ be a symplectic matrix over *V*. Assume that $U \subseteq V$ is an *A*-invariant subspace on which *A* acts irreducibly, and assume that *U* is not isotropic. Then *U* is symplectic, and its orthogonal complement $U^{\perp }$ is also *A*-invariant and symplectic.

#### Proof

Assume for contradiction that the restriction of the above bilinear form $\langle \cdot , \cdot \rangle$ to *U* is degenerate. Then there exist nonzero ${\textbf{u}}_{0} \in U$ such that $\langle {\textbf{u}}, {\textbf{u}}_{0} \rangle = 0$ for all ${\textbf{u}}\in U$, and hence $\langle A^{i}{\textbf{u}}, A^{i}{\textbf{u}}_{0} \rangle = 0$ for all ${\textbf{u}}\in U$ and all integers $i \ge 0$ (note that here we follow the usual convention of groups acting on the left.)

Since *A* is symplectic it is invertible, and so is the restriction to *U*, hence $\langle {\textbf{u}}, A^{i}{\textbf{u}}_{0} \rangle = 0$ for all ${\textbf{u}}\in U$. Since the span of $A^i{\textbf{u}}_{0}$, $i =0,1, \ldots$, equals *U* we find that $U \subseteq U^{\perp }$, contradicting that *U* is not isotropic.

The argument for the first part of second assertion is similar. If $\vec {w}\in U^{\perp }$ then $\langle {\textbf{u}},\vec {w}\rangle = 0$ for all ${\textbf{u}}\in U$, and thus $\langle A{\textbf{u}},A\vec {w}\rangle = 0$ for all ${\textbf{u}}\in U$ and hence, again using that $A|_{U}$ (that is, the map induced by *A* on *U*) is invertible, we have $\langle {\textbf{u}},A\vec {w}\rangle = 0$ for all ${\textbf{u}}\in U$ and thus $U^{\perp }$ is *A*-invariant. Since *U* is symplectic we have $U \cap U^{\perp } =\{0\}$ and thus $W = U \oplus U^{\perp }$ (since $\dim (U)+\dim (U^{\perp }) = \dim (W)$ always holds).

Now, if the restriction of the form to $U^{\perp }$ is degenerate there exists ${\textbf{v}}\in U^{\perp }$ with $\langle {\textbf{v}}, {\textbf{u}}^{\perp } \rangle = 0$ for all ${\textbf{u}}^{\perp } \in U^{\perp }$, and since $\langle {\textbf{v}},{\textbf{u}}\rangle = 0$ for all ${\textbf{u}}\in U$, we find that $\langle {\textbf{v}}, \vec {w}\rangle = 0$ for all $\vec {w}\in W$, which contradicts *W* being symplectic. $\square$

A simple consequence of Lemma 6.1 is that if *W* splits into irreducible *A*-invariant subspaces, then each such subspace is either symplectic or isotropic. If there exist an invariant isotropic subspace, there is scarring as shown by Kelmer [16, Theorem 1]. Otherwise, we can decompose *W* into smaller invariant symplectic subspaces and use a certain tensor product structure to reduce the dimension, and this allows us to treat the problem of small zero-divisors.

### Quantized cat maps and tensor products revisited

Let $A\in \operatorname {Sp}(2g,{{\mathbb {Z}}})$ have separable characteristic polynomial and let $N=p$ be a prime. Let us consider an element $\vec {n}\in {{\mathbb {Z}}}^{2g}$ for which the reduction modulo *p* in ${{\mathbb {Z}}}^{2g}/(p {{\mathbb {Z}}}^{2g}) \simeq {\mathbb {F}}_p^{2g}$ is not a zero-divisor in the sense defined in § 4.2, where we identify ${\mathbb {F}}_p^{2g} \simeq {\mathbb {F}}_p[x]/(f_{A}(x))$. In order to bound the matrix coefficient $\langle \operatorname {T}_N^{(r)}(\vec {n})\psi ,\psi ' \rangle$ we need some further properties of the quantization related to invariant symplectic subspaces and an associated tensor product structure; these properties are consequences of $U_{p,r}(A)$ being implicitly defined via the Weil (or oscillator) representation of $\operatorname {Sp}(2g,{{\mathbb {F}}}_{p})$. We briefly outline the construction below, for more details see [10, 16].

Hereafter, to simplify the notation in this section we regard *p* as a fixed prime, and suppress the dependence on *p* and $\vec {n}$ in most places. Let *W* be a symplectic vector space over ${{\mathbb {F}}}_p$, and assume that *W* splits into a direct sum of symplectic subspaces, that is, $W = W_{1} \oplus W_{2}$ where $W_{1} \perp W_{2}$ (that is, $W_2=W_1^{\perp }$), and the restrictions of the symplectic form to $W_{1}$ and $W_{2}$ are both non-degenerate.

We emphasise that in our application, $W_{1},W_{2}$ depend not only on *p* but on $\vec {n}$ as well: we write ${\mathbb {F}}_p^{2g} \simeq W_{1} \oplus W_{2}$, where the image of $\vec {n}$ in $W_{2}$ is zero, whereas the image in $W_{1}$ does not correspond to a zero-divisor.

With $V_{i} \subseteq W_{i}$, $i=1,2$, denoting maximal isotropic subspaces, we note that $V = V_{1} \oplus V_{2} \subseteq W$ is a maximal isotropic subspace. We may define the Heisenberg group

$$\begin{aligned} H(W) = \{ (f,\vec {w}):~f \in {{\mathbb {F}}}_p,\ \vec {w}\in W\} \end{aligned}$$

with the group law given by

$$\begin{aligned} (f,\vec {w}) \cdot (f',\vec {w}') = (f+f'+\langle \vec {w}, \vec {w}'\rangle , \vec {w}+\vec {w}') \end{aligned}$$

where $\langle \cdot , \cdot \rangle$ denotes the symplectic form on *W* (and similarly $H(W_{i})$ for $i=1,2$).

Let $Z \subseteq H(W_{1}) \times H(W_{2})$ denote the subgroup

$$\begin{aligned} Z = \{ (f, {{\textbf{0}}}) \times (-f, {{\textbf{0}}}):~f \in {{\mathbb {F}}}_p \}. \end{aligned}$$

We find that the surjection $H(W_{1}) \times H(W_{2}) \rightarrow H(W)$, given by

$$\begin{aligned} (f_{1},\vec {w}_{1}) \times (f_{2},\vec {w}_{2}) \rightarrow (f_{1}+f_{2}, \vec {w}_{1}+\vec {w}_{2}) \end{aligned}$$

factors through *Z*, and that we have the isomorphism

$$\begin{aligned} (H(W_{1}) \times H(W_{2}))/Z \cong H(W). \end{aligned}$$

The irreducible non-abelian representations of *H*(*W*) arise in the following way. Given a non-trivial additive character $\chi : {{\mathbb {F}}}_p \rightarrow {{\mathbb {C}}}$, let $K = {{\mathbb {F}}}_p \times V \subseteq H(W)$ denote a maximal abelian subgroup of *H*(*W*) and extend $\chi$ to *K* (say, by letting $\chi (f,v) = \chi (f)$). We remark that the character $\chi$ depends on *r* present in the definition of our observables $T_{p}^{(r)}$, but the precise dependence is not important; we only need that $\gcd (r,p) = 1$ implies that $\chi$ is non-trivial. By inducing the extended character $\chi$ from *K* to *H*(*W*), we obtain an irreducible representation $\rho :~H(W) \rightarrow GL( L^{2}(V))$, and similarly irreducible representations

$$\begin{aligned} \rho _{\nu }:~H(W_{\nu }) \rightarrow GL( L^{2}(V_{\nu })), \qquad \nu =1,2. \end{aligned}$$

Now, as $V = V_{1} \times V_{2}$ we have $L^{2}(V) = L^{2}(V_{1}) \otimes L^{2}(V_{2})$. Since the action of *Z* is trivial, we find that $H(W_{1}) \times H(W_{2})$, and thus *H*(*W*), acts in a natural way on $L^{2}(V_{1}) \otimes L^{2}(V_{2})$.

Briefly, the Weil representation $\pi$ of $\operatorname {Sp}(2g, {{\mathbb {F}}}_p)=\operatorname {Sp}(W)$ is then defined as follows: $\operatorname {Sp}(W)$ acts on *H*(*W*), and this induces an action on the set of irreducible representations of *H*(*W*). The action preserves the central character, and since irreducible representations of *H*(*W*) are determined by their central characters (this holds since *H*(*W*) is a two step nilpotent group), the action on the set of irreducible representations is, up to intertwining operators, trivial. In particular, for each $g \in \operatorname {Sp}(W)$, define $\rho ^{g}$ by $\rho ^{g}(h) = \rho ( g(h))$ (for $h \in H(W)$); we then find that $\rho \simeq \rho ^{g}$, that is, there exists an intertwining operator (only defined up to a scalar; it turns out that this gives projective representation of $\operatorname {Sp}(W)$; for *p* odd a non-trivial fact is that it is possible to choose scalars to obtain a true representation) $\pi (g)$ acting on $L^{2}(V) = L^{2}(V_{1}) \otimes L^{2}(V_{2})$ so that $\pi (g) \rho ^{g} = \rho \pi (g)$. Further, we similarly obtain “smaller” Weil representations $\rho _{\nu }$ of $\operatorname {Sp}(W_{\nu })$ acting on $L^{2}(V_{\nu })$, for $\nu =1,2$; to fix compatible central characters it is convenient to use the maps $H(W_{\nu }) \rightarrow H(W_{1}) \times H(W_{2}) \rightarrow H(W)$ to obtain the action of $\operatorname {Sp}(W_\nu )$ on $L^{2}(V_{\nu })$.

The product $\operatorname {Sp}(W_{1}) \times \operatorname {Sp}(W_{2})$, under the inclusion

$$\begin{aligned}\operatorname {Sp}(W_{1}) \times \operatorname {Sp}(W_{2}) \subseteq \operatorname {Sp}(W), \end{aligned}$$

then acts componentwise on the tensor product $L^{2}(V_{1}) \otimes L^{2}(V_{2})$. In particular, if $A \in \operatorname {Sp}(W)$ leaves both $W_{1}$ and $W_{2}$ invariant, let $A_{\nu } \in \operatorname {Sp}(W_{\nu })$ denote the corresponding restrictions of *A* to $W_{\nu }$, for $\nu =1,2$. We now note that letting $\vec {w}=\vec {w}_{1}+\vec {w}_{2}$ denote the reduction of $\vec {n}$ modulo *p*, we can write,

6.1 $$\begin{aligned} \begin{aligned}&U_{p,r}(A) = U_{1} (A_{1}) \otimes U_{2}(A_{2}), \\&\operatorname {T}_p^{(r)}(\vec {n}) = \rho ((0,\vec {w})) = \rho _{1}((0,\vec {w}_{1})) \otimes \rho _{2}((0,\vec {w}_{2})), \end{aligned} \end{aligned}$$

where $U_{p,r}(A) = \pi (A)$ and $U_{\nu }(A_{\nu }) = \pi _{\nu }(A_{\nu })$ for $\nu =1,2$.

### Eigenfunctions of tensor products

We next describe eigenfunctions of $U_{p,r}(A)$ in terms of the tensor product structure. With $W,W_1,W_2$ and $V,V_{1},V_{2}$ as in § 6.3, for $\nu =1,2$, we may decompose $L^{2}(V_{\nu })$ into $U_{\nu }(A_{\nu })$-eigenspaces

$$\begin{aligned} E_{\nu ,\lambda } = \ker ( U_{\nu }(A_{\nu }) - \lambda I), \qquad \lambda \in \Lambda _{\nu }, \end{aligned}$$

(possibly with multiplicities), where $\lambda$ ranges over the set of eigenvalues $\Lambda _{\nu }$ of $U_{\nu }(A_{\nu })$.

Further, for $\nu =1,2$ we may find bases of orthonormal eigenfunctions $\psi _{\nu ,\lambda ,i} \in E_{\nu ,\lambda }$, $i = 1, \ldots , I_{\nu ,\lambda }$, for some positive integers $I_{\nu ,\lambda }= \dim (E_{\nu ,\lambda })$ with

$$\begin{aligned} \sum _{\lambda \in \Lambda _{1}} I_{1,\lambda } + \sum _{\lambda \in \Lambda _{2}} I_{2,\lambda }= 2g, \end{aligned}$$

which follows from the separability of the characteristic polynomial of *A*. That is,

$$\begin{aligned} U_{\nu }(A_{\nu }) \psi _{\nu ,\lambda ,i} = \lambda _{\nu ,i} \psi _{\nu ,\lambda ,i}, \qquad \nu = 1,2, \ i = 1, \ldots , I_{\nu ,\lambda }, \end{aligned}$$

where $\lambda _{\nu ,i}$ ranges over the whole set $\Lambda _{\nu }$. We further note that the set

$$\begin{aligned} \{ \psi _{1,\lambda _{1},i_1} \otimes \psi _{2,\lambda _{2},i_2}:~ \lambda _{\nu } \in \Lambda _\nu , \ i_\nu = 1, \ldots , I_{\nu ,\lambda }, \ \nu =1,2\} \end{aligned}$$

gives an orthonormal eigenbasis of $L^{2}(V) = L^{2}(V_{1}) \otimes L^{2}(V_{2})$. In particular, the eigenvalues of $U_{p,r}(A) = U_{1}(A_{1}) \otimes U_{2}(A_{2})$ are given by

$$\begin{aligned} \Lambda = \{ \lambda _{1} \lambda _{2}:~\lambda _{1} \in \Lambda _{1}, \lambda _{2} \in \Lambda _{2}\}, \end{aligned}$$

and for $\mu \in \Lambda$, an eigenbasis for $E_{\mu } = \ker ( U_{p,r}(A) - \mu I)$ is given by

$$\begin{aligned} \{ \psi _{1,\lambda ,i} \otimes \psi _{2,\mu /\lambda ,j}:~ \lambda \in \Lambda _1, \ i = 1, \ldots , I_{1,\lambda }, \ j = 1, \ldots , I_{2,\mu /\lambda } \}. \end{aligned}$$

Note that the quantizations $U_{p,r}(A), U_{1}(A_{1})$, and $U_{2}(A_{2})$ are only defined up to scalars, but once we have chosen scalars for $U_{1}(A_{1})$, and $U_{2}(A_{2})$ we may chose the scalar for $U_{p,r}(A)$ so that multiplicativity of eigenvalues hold.

We can now bound matrix coefficients corresponding to observables having zero-divisors.

#### [Style2 Style2 Style2]Lemma 6.2

Let $$A\in \operatorname {Sp}(2g,{{\mathbb {Z}}})$$ with a separable characteristic polynomial, such that there are no *A*-invariant rational istropic subspaces. There exists some constant $$\gamma >0$$, depending only on *A*, such that for a positive proportion of primes *p* the following holds: Let $$\psi \in E_{\mu }$$ and $$\psi ' \in E_{\mu '}$$ denote two eigenfunctions of $$U_{p,r}(A) $$, and let $$\vec {w}$$ denote a non-trivial zero-divisor. Then for $$p > p_0(A) \Vert \vec {w}\Vert _2^{2g}$$, where $$p_0(A)$$ is as in Lemma 4.4, we have

$$\begin{aligned} |\langle \operatorname {T}_p^{(r)}(\vec {w}) \psi ,\psi '\rangle | \ll p^{-\gamma } \Vert \psi \Vert _{2} \cdot \Vert \psi ' \Vert _{2}. \end{aligned}$$

#### Proof

Let ${{\textbf{0}}}\ne \vec {w}\in {{\mathbb {Z}}}^{2g}$, which is a zero-divisor. Then there is an *A*-stable rational subspace $W_1$, necessarily symplectic by Lemma 6.1, so that with respect to the decomposition $\vec {w}= (\vec {w}_{1},\vec {w}_{2}) \in W_1\oplus W_2$, where $W_2= W_1^\perp$, the component $\vec {w}_1\in W_1$ of $\vec {w}$ is not a zero divisor, while the component $\vec {w}_{2}$ in $W_2=W_1^\perp$ is zero.

For $$\mu , \mu ' \in \Lambda $$, write

$$\begin{aligned} \psi = \sum _{(\lambda , i, j) \in \Omega } \alpha _{\lambda ,i,j} \psi _{1,\lambda ,i} \otimes \psi _{2,\mu /\lambda ,j}, \end{aligned}$$

where

$$\begin{aligned} \Omega = \{(\lambda , i, j):~ \lambda \in \Lambda _1, \ i = 1, \ldots , I_{1,\lambda }, \ j = 1, \ldots , I_{2,\mu /\lambda }\}, \end{aligned}$$

and

$$\begin{aligned} \psi ' = \sum _{(\lambda ', i', j')\in \Omega '} \beta _{\lambda ',i',j'} \psi _{1,\lambda ',i'} \otimes \psi _{2,\mu '/\lambda ',j'}, \end{aligned}$$

where

$$\begin{aligned} \Omega ' = \{(\lambda ', i', j'):~ \lambda ' \in \Lambda _1, \ i' = 1, \ldots , I_{2,\lambda '}, \ j' = 2, \ldots , I_{2,\mu '/\lambda '}\}, \end{aligned}$$

with complex coefficients $\alpha _{\lambda ,i,j}, \beta _{\lambda ',i',j'} \in {{\mathbb {C}}}$.

Since $\vec {w}_{2}={{\textbf{0}}}$, by (6.1), we have

$$\begin{aligned} \operatorname {T}_p^{(r)}(\vec {w})=\rho ((0,\vec {w}))= \rho _{1}((0,\vec {w}_{1})) \otimes \rho _{2}((0,\vec {w}_{2})) = \rho _{1}((0,\vec {w}_{1})) \otimes \textrm{Id}, \end{aligned}$$

and thus

$$\begin{aligned} \langle \rho ((0,\vec {w})) \psi , \psi '\rangle = \sum _{(\lambda , i, j) \in \Omega } \sum _{(\lambda ', i', j')\in \Omega '}&\overline{\alpha _{\lambda ,i,j}} \beta _{\lambda ',i',j'} \\ \cdot \langle \rho _1((0,\vec {w}_1))&\psi _{1,\lambda ,i},\psi _{1,\lambda ',i'} \rangle \langle \psi _{2,\mu /\lambda ,j}, \psi _{2,\mu '/\lambda ',j'} \rangle . \end{aligned}$$

Now, since

$$\begin{aligned} \langle \psi _{2,\mu /\lambda ,j}, \psi _{2,\mu '/\lambda ',j'} \rangle = {\left\{ \begin{array}{ll} 1& \text {if }j=j'\text { and }\mu /\lambda = \mu '/\lambda ',\\ 0& \text {otherwise,} \end{array}\right. } \end{aligned}$$

only terms for which $j=j'$ and for which $\lambda ' = \eta (\lambda )$ for the bijection $\eta : \Lambda _{1} \rightarrow \Lambda _{1}$ contribute (more precisely, we have $\eta (\lambda ) = (\lambda \mu ')/\mu$. Hence

6.2 $$\begin{aligned} \begin{aligned}&\langle \rho ((0,\vec {w})) \psi , \psi '\rangle \\&\quad = \sum _{(\lambda ,i,j) \in \Omega } \sum _{i'=1}^{I_{1, \eta (\lambda )}} \overline{\alpha _{\lambda ,i,j}} \beta _{\eta (\lambda ),i',j} \langle \rho _1((0,\vec {w}_1)) \psi _{1,\lambda ,i},\psi _{1,\eta (\lambda ),i'} \rangle . \end{aligned} \end{aligned}$$

We now apply Corollary 5.4 with respect to the matrix $A_1$ in the decomposition (6.1), which applies since $\vec {w}_{1}$ is not a zero-divisor. Then, by the Cauchy-Schwarz inequality, for every $\lambda$ and *j* fixed, we have

6.3 $$\begin{aligned} \begin{aligned}&\left| \sum _{i=1}^{I_{1, \lambda }} \sum _{i'=1}^{I_{1, \eta (\lambda )}} \overline{\alpha _{\lambda ,i,j}} \beta _{\eta (\lambda ),i',j} \langle \rho _1((0,\vec {w}_1)) \psi _{1,\lambda ,i},\psi _{1,\eta (\lambda ),i'} \rangle \right| \\&\quad \ll p^{-\gamma } \left( \sum _{i=1}^{I_{1, \lambda }}|\alpha _{\lambda ,i,j}|^{2} \right) ^{1/2} \left( \sum _{i'=1}^{I_{1, \eta (\lambda )}} |\beta _{\eta (\lambda ),i',j}|^{2}\right) ^{1/2} \end{aligned} \end{aligned}$$

Finally, using the Cauchy-Schwarz inequality again, and then recalling that $\eta$ is a bijection on $\Lambda$, we derive

$$\begin{aligned}&\sum _{\lambda \in \Lambda } \sum _{j=1}^{I_{2, \mu /\lambda }} \left( \sum _{i=1}^{I_{1, \lambda }}|\alpha _{\lambda ,i,j}|^{2} \right) ^{1/2} \left( \sum _{i'=1}^{I_{1, \eta (\lambda )}} |\beta _{\eta (\lambda ),i',j}|^{2}\right) ^{1/2} \\&\quad \ll \left( \sum _{\lambda \in \Lambda } \sum _{j=1}^{I_{2, \mu /\lambda }} \sum _{i=1}^{I_{1, \lambda }}|\alpha _{\lambda ,i,j}|^{2} \right) ^{1/2} \left( \sum _{\lambda \in \Lambda } \sum _{j=1}^{I_{2, \mu /\lambda }} \sum _{i'=1}^{I_{1, \eta (\lambda )}} |\beta _{\eta (\lambda ),i',j}|^{2} \right) ^{1/2} \\&\quad = \Vert \psi \Vert _{2} \cdot \Vert \psi ' \Vert _{2} \end{aligned}$$

and recalling (6.2) and (6.3), we conclude the proof. $\square$

## Anatomy of Integers

### Some sums and products over primes

It is convenient to denote by $\log _{k} x$ the *k*-fold iterated logarithm, that is, for $x\geqslant 1$ we set

$$\begin{aligned} \log _1 x = \log x \qquad \text{ and } \qquad \log _k = \log _{k-1} \max \{\log x, 2\}, \quad k =2, 3, \ldots . \end{aligned}$$

We begin by recording an upper bound for Mertens type sums over primes in progressions, together with a simple consequence.

#### [Style2 Style2 Style2]Lemma 7.1

Let *q* be a prime and let $j \geqslant 1$ be an integer. We have

$$\begin{aligned} \sum _{\begin{array}{c} p \leqslant x\\ q \mid p^{j} -1 \end{array} } \frac{1}{p} \ll q^{-1/j} + \frac{\log _2 x }{q}, \end{aligned}$$

where the implied constant depends only on *j*.

#### Proof

For an integer $k \ge 0$ define the dyadic interval $I_{k} = [ 2^{k}q, 2^{k+1}q ]$, and note that $q\mid p^{j}-1$ implies that *p* must lie in a progression $p \equiv a \,\operatorname {mod}q$, where $0 \leqslant a < q$ ranges over over at most *j* possible values. For any *a*, the Brun–Titchmarsh inequality, see, for example, [14, Theorem 6.6] or [27, Chapter I, Theorem 4.16], implies that

$$\begin{aligned} \sum _{\begin{array}{c} p \in I_{k} \\ p \equiv a \,\operatorname {mod}q \end{array} } 1/p \ll \frac{2^{k+1}q }{ q \log ( 2^{k+1}q/q) } \cdot \frac{1}{2^{k}q} \ll \frac{1}{q (k+1)}. \end{aligned}$$

If $2^{k}q \leqslant x$ we have $k \ll \log x$, and summing over such *k* we find that the contribution from primes $p \geqslant q$ is $O\left( q^{-1} \log _{2}x\right)$. Since there are at most *j* primes $p < q$ for which $q \mid p^{j}-1$, and each such prime satisfies $p > q^{1/j}$ we find that the contribution from $p < q$ is $O\left( q^{-1/j}\right)$, and the proof is concluded. $\square$

We remark that for $j=1$ the bound of Lemma 7.1 simplifies as

7.1 $$\begin{aligned} \sum _{\begin{array}{c} p \leqslant x\\ p \equiv 1 \bmod q \end{array} } \frac{1}{p} \ll \frac{\log _2 x }{q}. \end{aligned}$$

We control the contribution from small prime divisors of $p -1$ as follows. For a prime *q* and positive integer *k*, we define $v_q(k)$ to be the positive integer $\ell$ such that

$$\begin{aligned} q^\ell \mid k \qquad \text{ and } \qquad q^{\ell +1} \not \mid k. \end{aligned}$$

We fix some $z > 0$ and let

7.2 $$\begin{aligned} s_z(N) = \prod _{p \mid N} \prod _{q \leqslant z} q^{v_q(p-1)} = \prod _{p \mid N} \prod _{\begin{array}{c} q \leqslant z\\ q^{\ell } \Vert p -1 \end{array}} q^{\ell }, \end{aligned}$$

that is, $s_z(N)$ is the product of the *z*-smooth parts of $p-1$, as *p* ranges over all prime divisors of *N*.

#### [Style2 Style2 Style2]Lemma 7.2

Let

$$\begin{aligned} Z = \exp \left( (\log _{2} x) (\log _{3} x)^{3/2}\right) \qquad \text{ and } \qquad z = \left( \log _2 x\right) ^{O(1)}. \end{aligned}$$

For all but *o*(*x*) integers $N \leqslant x$ we have $s_z(N) \leqslant Z$.

#### Proof

From the definition of $s_z(N)$ in (7.2), extending over all powers $q^\ell \leqslant x$, $q\leqslant z$, such that $q^\ell \mid (p-1)$, we have

$$\begin{aligned} \sum _{N \leqslant x} \log s_z(N) \ll \sum _{\begin{array}{c} q^{\ell } \leqslant x\\ q \leqslant z,~\textrm{prime} \end{array}} \log (q^{\ell }) \sum _{p \equiv 1 \,\operatorname {mod}q^{\ell }} \left\lfloor x/p\right\rfloor = S_1 + S_{\geqslant 2}, \end{aligned}$$

where $S_1$ is the contribution from the terms corresponding to $\ell =1$ and $S_{\geqslant 2}$ is the contribution from the terms with $\ell \geqslant 2$.

For $S_1$, we have

$$\begin{aligned} S_1 \ll x \sum _{q \leqslant z,~\textrm{prime} } \log q \sum _{\begin{array}{c} p\leqslant x\\ p \equiv 1 \,\operatorname {mod}q \end{array}} \frac{1}{p}. \end{aligned}$$

Using (7.1) applied to the inner sum, we now derive

7.3 $$\begin{aligned} \begin{aligned} S_1&\ll x \sum _{q \leqslant z} \log q \frac{\log _{2} x}{q} \\&\ll x (\log _2 x) \sum _{q \leqslant z} \frac{\log q}{q} \ll x (\log _2 x) (\log z). \end{aligned} \end{aligned}$$

The sum $S_{\geqslant 2}$ is estimated trivially by discarding the primality conditions on *p* and thus using that

$$\begin{aligned} \sum _{\begin{array}{c} p\leqslant x\\ p \equiv 1 \,\operatorname {mod}q^{\ell } \end{array}} \frac{1}{p} \leqslant \sum _{1 \leqslant k \leqslant x/q^{\ell }} \frac{1}{1+k q^\ell } \ll \frac{\log x}{q^\ell }, \end{aligned}$$

which implies, after we abandon the condition of primality on *q* and the inequality $q \leqslant z$,

7.4 $$\begin{aligned} \begin{aligned} S_{\geqslant 2}&\ll x(\log x) \sum _{2 \leqslant \ell \leqslant \log x/\log 2} \, \sum _{1\leqslant m \leqslant x^{1/\ell }} \frac{\log (m^{\ell })}{m^\ell } \\&\ll x (\log x)^2 \sum _{2 \leqslant \ell \leqslant \log x/\log 2} x^{-1 +1/\ell }\ll x^{1/2} (\log x)^2. \end{aligned} \end{aligned}$$

Clearly the bound on $S_1$ in (7.3) dominates the bound on $S_{\geqslant 2}$ in (7.4). Hence,

$$\begin{aligned} \sum _{N \leqslant x} \log s_z(N) \ll x (\log _2 x) (\log z) \ll x (\log _2 x) (\log _3 x). \end{aligned}$$

Therefore we have $s_z(N) \geqslant Z = \exp \left( (\log _{2} x) (\log _{3} x)^{3/2}\right)$ for at most

$$\begin{aligned} O\left( x (\log _2 x) (\log _3 x) (\log Z)^{-1} \right) = O\left( x\left( \log _3 x\right) ^{-1/2}\right) \end{aligned}$$

positive integers $$N \leqslant x$$. $$\square $$

### Good primes and integers

We recall that $A \in \operatorname {Sp}(2g,{{\mathbb {Z}}})$.

We say that a prime *p* is *good* if the following two conditions are satisfied:

- the characteristic polynomial of *A* is separable and splits completely modulo *p*;
- for the roots $\lambda _1, \ldots , \lambda _{2g}$ of the characteristic polynomial of *A* modulo *p* we have
  $$\begin{aligned} \operatorname {ord}(\lambda _i,p),\, \operatorname {ord}(\lambda _i/\lambda _j,p) \geqslant p^{1/3}, \qquad 1\leqslant i, j\leqslant s, \ i \ne j. \end{aligned}$$

We note that the exponent 1/3 is somewhat arbitrary and can be replaced by any $\gamma < 1/2$.

Let ${{\mathcal {P}}}_{\textrm{good}}$ denote the set of good primes.

Applying Lemma 5.1, we now derive

#### [Style2 Style2 Style2]Lemma 7.3

The set ${{\mathcal {P}}}_{\textrm{good}}$ is of positive density.

Next, given integers $U \geqslant V \geqslant 1$ we define

$$\begin{aligned} {{\mathcal {P}}}_{\textrm{good}}(V,U) = {{\mathcal {P}}}_{\textrm{good}}\cap [V,U]. \end{aligned}$$

We now set

7.5 $$\begin{aligned} \begin{aligned}&D(x) = ( \log x)^{(\log _{3} x)^{2}},\\&V(x) = \exp \left( \exp \left( \sqrt{\log _2 x}\right) \right) , \\&W(x) = x^{\log _{3}x/\log _{2} x} , \\ \end{aligned} \end{aligned}$$

and define the following set ${{\mathcal {N}}}_{\textrm{good}}(x)$ of *good* integers

7.6 $$\begin{aligned} \begin{aligned} {{\mathcal {N}}}_{\textrm{good}}= \{ N :~\exists p&\in {{\mathcal {P}}}_{\textrm{good}}(V(N),W(N)) \\&\text {with } N = p M, \ M \in {{\mathbb {Z}}},\ \gcd (p, M) =1, \\&\qquad \qquad \gcd \left( p-1,\operatorname {ord}(A,M)\right) \leqslant D(N)\}. \end{aligned} \end{aligned}$$

We then set

$$\begin{aligned} {{\mathcal {N}}}_{\textrm{good}}(x) = {{\mathcal {N}}}_{\textrm{good}}\cap [1,x]. \end{aligned}$$

The next statement is our main tool.

#### [Style2 Style2 Style2]Lemma 7.4

We have

$$\begin{aligned} \sharp {{\mathcal {N}}}_{\textrm{good}}(x) = x + o(x). \end{aligned}$$

#### Proof

It is certainly enough to show that

$$\begin{aligned}\sharp \left( {{\mathcal {N}}}_{\textrm{good}}\cap [x/2,x]\right) = x/2 + o(x). \end{aligned}$$

In turn, we set

$$\begin{aligned} D_0 = D(x/2), \quad V_0 = V(x), \quad W_0 = W(x/2), \end{aligned}$$

such that

$$\begin{aligned} {[}V_0,W_0]\subseteq [V(N),W(N)], \end{aligned}$$

for all $N \in [x/2, x]$ and define the following set ${{\widetilde{{{\mathcal {N}}}}}}_{\textrm{good}}(x)$ of *good* integers $N \leqslant x$:

$$\begin{aligned} {{\widetilde{{{\mathcal {N}}}}}}_{\textrm{good}}(x) = \{ N \leqslant x&:~\exists p\in {{\mathcal {P}}}_{\textrm{good}}(V_0,W_0) \text { with }N = p M, \ M \in {{\mathbb {Z}}}, \\&\quad \gcd (p, M) =1, \ \gcd \left( p-1,\operatorname {ord}(A,M)\right) \leqslant D_0\}. \end{aligned}$$

Clearly

$$\begin{aligned} {{\widetilde{{{\mathcal {N}}}}}}_{\textrm{good}}(x) \cap [x/2,x] \subseteq {{\mathcal {N}}}_{\textrm{good}}\cap [x/2,x], \end{aligned}$$

hence it is enough to show that

7.7 $$\begin{aligned} \sharp {{\widetilde{{{\mathcal {N}}}}}}_{\textrm{good}}(x) = x + o(x). \end{aligned}$$

That is, in the above, we first consider integers *N* in a dyadic interval. This allows us to replace ${{\mathcal {P}}}_{\textrm{good}}(V(N),W(N))$ with ${{\mathcal {P}}}_{\textrm{good}}(V_0,W_0)$. After this is done, we can bring back integers below *x*/2 as well: if the exceptional set is of size *o*(*x*) on [1, *x*] then so it is on [*x*/2, *x*] and we are done. Thus indeed we only need to establish (7.7).

First recall that by Lemma 7.3 the set of good primes ${{\mathcal {P}}}_{good}$ is of positive density. Therefore, there are some constants $C, c>0$ (depending on the matrix *A*) such that for $Z \geqslant 2$ the set ${{\mathcal {P}}}_{\textrm{good}}(Z, CZ)$ contains at least $cZ/\log Z + O(1)$ primes, that is,

7.8 $$\begin{aligned} \sharp {{\mathcal {P}}}_{\textrm{good}}(Z, CZ) \geqslant c\frac{Z}{\log Z} + O(1). \end{aligned}$$

Taking *x* sufficiently large such that the interval $$[2,W_0]$$ contains *I* non-overlapping intervals of the form $$[C^i, C^{i+1} )$$, $$i =1, \ldots , I$$, where

$$\begin{aligned} \log W_0 \ll I \ll \log W_0, \end{aligned}$$

we derive

$$\begin{aligned} \sum _{p \in {{\mathcal {P}}}_{\textrm{good}}\left( V_0,W_0\right) } 1/p&\geqslant \sum _{p \in {{\mathcal {P}}}_{\textrm{good}}(2,W_0)} 1/p - \sum _{p \leqslant V_0,~\textrm{prime}}1/p \\&\geqslant \sum _{i=1}^I \sum _{p \in {{\mathcal {P}}}_{\textrm{good}}\left( C^i , C^{i+1}\right) } 1/p - \sum _{p \leqslant V_0,~\textrm{prime}}1/p \\&\geqslant \sum _{i=1}^I C^{-i} \sharp {{\mathcal {P}}}_{\textrm{good}}\left( C^i , C^{i+1}\right) - \sum _{p \leqslant V_0,~\textrm{prime}}1/p . \end{aligned}$$

Next, recalling (7.8) and the Mertens formula (or simply using (7.1) with $q =1$), we obtain

$$\begin{aligned} \sum _{p \in {{\mathcal {P}}}_{\textrm{good}}\left( V_0,W_0\right) } 1/p&\geqslant \sum _{i=1}^I C^{-i} \left( c\frac{C^i}{i \log C} + O(1)\right) + O(\log _2 V_0) \\&\geqslant \frac{c}{\log C} \sum _{i=1}^I \frac{1}{i} + O(\log _2 V_0) \geqslant \frac{c}{\log C} \log I+ O(\log _2 V_0) \\&\gg \log _{2} W_0 + O(\log _2 V_0) \gg \log _{2} W_0 \gg \log _{2} x. \end{aligned}$$

Therefore,

$$\begin{aligned} \prod _{p \in {{\mathcal {P}}}_{\textrm{good}}\left( V_0,W_0\right) } (1-1/p) \ll \exp \left( - \ \sum _{p \in {{\mathcal {P}}}_{\textrm{good}}\left( V_0,W_0\right) } 1/p \right) \leqslant (\log x)^{-\gamma } \end{aligned}$$

for some $\gamma >0$, which depends only on *C* and *c*, and thus only on the matrix *A*. Thus, by the classical Brun sieve, see, for example, [27, Chapter I, Theorem 4.4], almost all $N \leqslant x$ are divisible by some prime $p \in {{\mathcal {P}}}_{\textrm{good}}\left( V_0,W_0\right)$.

We now set $z = \left( \log _2 x\right) ^{2g+1}$ and note that $D_0 > Z$, where *Z* is as in Lemma 7.2. Thus Lemma 7.2 allows us to discard *o*(*x*) positive integers $N \leqslant x$ with

$$\begin{aligned} s_z(N) \geqslant D_0, \end{aligned}$$

where $s_z(N)$ is defined by (7.2). Hence for the remaining integers $N \in [x/2,x]$ we have

$$\begin{aligned} s_z(N) < D_0 \leqslant D(N). \end{aligned}$$

We also discard $O(x/V_0)$ integers $N\leqslant x$ which are divisible by $p^2$ for some prime $p > V_0$. Hence, for the remaining integers *N*, for any $p\in {{\mathcal {P}}}_{\textrm{good}}(V_0,W_0)$ with $p \mid N$ we now have $\gcd (p, N/p) =1$.

Furthermore, for the remaining $N \leqslant x$, we see that if

$$\begin{aligned} \gcd \left( p-1,\operatorname {ord}(A,N/p)\right) >D_0, \end{aligned}$$

then, since $s_z(p)< s_z(N) < D_0$, there is a prime $q> z$ with $q\mid p-1$ and another prime $\ell \mid N$, $\ell \ne p$, such that

$$\begin{aligned} q \mid \operatorname {ord}(A,\ell ) \mid \prod _{j=1}^{2g} \left( \ell ^{j}-1\right) . \end{aligned}$$

Hence to conclude the proof it suffices to show that for every $j =1, \ldots , 2g$ we have

7.9 $$\begin{aligned} \sum _{\begin{array}{c} q > z, \ \textrm{prime} \end{array}}\ \sum _{\begin{array}{c} p \leqslant x, \ \textrm{prime} \\ p \equiv 1 \,\operatorname {mod}q \end{array}}\ \sum _{\begin{array}{c} \ell \leqslant x/p, \ \textrm{prime}\\ p \ne \ell \\ q \mid \ell ^j-1 \end{array}} \frac{x}{\ell p} = o(x). \end{aligned}$$

To establish (7.9), we first discard the condition $\ell \ne p$, and extend the summation over $\ell$ up to $\ell \leqslant x$. Then we recall Lemma 7.1 (for the sum over $\ell$) and its special case (7.1) (for the sum over *p*) and derive

$$\begin{aligned} \sum _{q> z, \ \textrm{prime}}\ \sum _{\begin{array}{c} p \leqslant x, \ \textrm{prime}\\ p \equiv 1 \,\operatorname {mod}q \end{array}} \ \sum _{\begin{array}{c} \ell \leqslant x/p, \ \textrm{prime} \\ p \ne \ell \\ q \mid \ell ^j-1 \end{array}} \frac{x}{\ell p}&\leqslant x \sum _{q> z,\ \textrm{prime}}\ \sum _{\begin{array}{c} p \leqslant x, \ \textrm{prime} \\ p \equiv 1 \,\operatorname {mod}q \end{array}}\frac{1}{p} \ \sum _{\begin{array}{c} \ell \leqslant x, \ \textrm{prime}\\ q \mid \ell ^j-1 \end{array}} \frac{1}{\ell } \\&\ll x \sum _{q>z, \ \textrm{prime} } \frac{\log _2 x }{q} \left( q^{-1/j} + \frac{\log _2 x }{q}\right) \\&\ll x\left( \frac{\log _2 x }{z^{1/j}} + \frac{(\log _2 x)^2 }{z^2}\right) \\&\ll x\left( \frac{\log _2 x }{z^{1/(2g)}} + \frac{(\log _2 x)^2 }{z^2}\right) . \end{aligned}$$

Recalling our choice $z = \left( \log _2 x\right) ^{2g+1}$, we obtain (7.9).

Thus all together we have discarded *o*(*x*) integers and all remaining integers $N\leqslant x$ belong to ${{\widetilde{{{\mathcal {N}}}}}}_{\textrm{good}}$. Hence we see that (7.7) holds, and the result follows. $\square$

## Proof of Theorem 1.2

We recall the definition of good integers given by (7.6). We now show that (1.7) holds with ${{\mathcal {N}}}= {{\mathcal {N}}}_{\textrm{good}}$, that is,

$$\begin{aligned} \lim _{\begin{array}{c} N\rightarrow \infty \\ N\in {{\mathcal {N}}}_{\textrm{good}} \end{array}}\max _{\psi _N,\psi '_N } \left| \langle \operatorname {Op}_N(f)\psi _{ N}, \psi _{N}' \rangle -\langle \psi _N,\psi _N'\rangle \int _{{\mathbb {T}}^{2g}} f({\textbf{x}})d{\textbf{x}}\right| = 0, \end{aligned}$$

where the maximum is taken over all pairs of normalized eigenfunctions $\psi _N,\psi '_N$ of $U_N(A)$. By Lemma 7.4, the set ${{\mathcal {N}}}_{\textrm{good}}$ is of full density and hence this is sufficient for our goal.

As in [2, 19], using the rapid decay of coefficients of $f\in C^\infty ({\mathbb {T}}^{2g})$, it suffices to show that

$$\begin{aligned} \max _{\begin{array}{c} \vec {n}\in {{\mathbb {Z}}}^{2g}\\ 0 < |\vec {n}| \leqslant L(N) \end{array}} \max _{ \psi _N,\psi _N' } \left| \langle \operatorname {T}_N(\vec {n}) \psi _N, \psi _N' \rangle \right| \rightarrow 0 \end{aligned}$$

as $N \rightarrow \infty$, $N\in {{\mathcal {N}}}_{\textrm{good}}$, with $\psi _N, \psi _N'$ running over all normalized eigenfunctions of $U_N(A)$, with a slowly growing function $L(N)\rightarrow \infty$.

We recall the definition of the functions *D*(*x*), *V*(*x*) and *W*(*x*) as in (7.5). In particular, we take *L*(*N*) to grow sufficiently slowly to guarantee that for any $p \in {{\mathcal {P}}}_{\textrm{good}}(V(N),W(N))$ and for any $\vec {n}\in {{\mathbb {Z}}}^{2g}$ with $0 < |\vec {n}| \leqslant L(N)$ the conditions of Corollary 5.4 and Lemma 6.2 are satisfied provided that *N* is sufficiently large.

We now fix some $N \in {{\mathcal {N}}}_{\textrm{good}}$ and choose a prime *p* which satisfies all properties in (7.6).

We set

$$\begin{aligned}d = \gcd \left( \operatorname {ord}(A,p), \operatorname {ord}(A, M )\right) .\end{aligned}$$

Clearly

$$\begin{aligned} d \leqslant \gcd (p - 1, \operatorname {ord}(A, M )) \leqslant D(N). \end{aligned}$$

Now, applying (3.6), and then Lemma 3.1 (with $N_1 = p$ and with $A^d$ instead of *A*), we derive

8.1 $$\begin{aligned} |\langle \operatorname {T}_N(\vec {n})\psi , \psi ' \rangle | \leqslant \max _{\varphi , \varphi ' \in \Phi _{p, r}} | \langle \operatorname {T}_{p}^{ r} (\vec {n})\varphi , \varphi ' \rangle |, \end{aligned}$$

where *r* is some integer coprime to *p* and $\varphi$, $\varphi '$ range over all normalized eigenfunctions of $U_{p, r}(A^d)$.

We note that the roots of the characteristic polynomial of $A^d$ are $\lambda _1^d, \ldots , \lambda ^d_{2g}$, where $\lambda _1, \ldots , \lambda _{2g}$ are the roots of the characteristic polynomial of *A* modulo *p* and we also have

$$\begin{aligned} \operatorname {ord}(\lambda _i^d,p),\, \operatorname {ord}(\lambda _i^d/\lambda _j^d,p) \geqslant p^{1/3} d^{-1} \gg p^{1/4}, \qquad 1\leqslant i, j\leqslant 2g, \ i \ne j, \end{aligned}$$

since obviously for a sufficiently large *N* we have

$$\begin{aligned} d\leqslant D(N) \leqslant V(N)^{1/12} \leqslant p^{1/12}. \end{aligned}$$

Thus the conditions of Corollary 5.3 are satisfied. Combining Corollary 5.4 and Lemma 6.2 (when $\vec {n}$ is a zero divisor) with (8.1) we conclude the proof.
